# Physiologic Data-Driven Iterative Learning Control for Left Ventricular Assist Devices

**DOI:** 10.3389/fcvm.2022.922387

**Published:** 2022-07-13

**Authors:** Konstantinos Magkoutas, Philip Arm, Mirko Meboldt, Marianne Schmid Daners

**Affiliations:** Product Development Group Zurich, Department of Mechanical and Process Engineering, ETH Zurich, Zurich, Switzerland

**Keywords:** LVAD, heart failure, data driven control, iterative learning control, VAD physiological control, ventricular assist devices, ILC, pulsatile blood pump

## Abstract

Continuous flow ventricular assist devices (cfVADs) constitute a viable and increasingly used therapy for end-stage heart failure patients. However, they are still operating at a fixed-speed mode that precludes physiological cfVAD response and it is often related to adverse events of cfVAD therapy. To ameliorate this, various physiological controllers have been proposed, however, the majority of these controllers do not account for the lack of pulsatility in the cfVAD operation, which is supposed to be beneficial for the physiological function of the cardiovascular system. In this study, we present a physiological data-driven iterative learning controller (PDD-ILC) that accurately tracks predefined pump flow trajectories, aiming to achieve physiological, pulsatile, and treatment-driven response of cfVADs. The controller has been extensively tested in an *in-silico* environment under various physiological conditions, and compared with a physiologic pump flow proportional-integral-derivative controller (PF-PIDC) developed in this study as well as the constant speed (CS) control that is the current state of the art in clinical practice. Additionally, two treatment objectives were investigated to achieve pulsatility maximization and left ventricular stroke work (LVSW) minimization by implementing copulsation and counterpulsation pump modes, respectively. Under all experimental conditions, the PDD-ILC as well as the PF-PIDC demonstrated highly accurate tracking of the reference pump flow trajectories, outperforming existing model-based iterative learning control approaches. Additionally, the developed controllers achieved the predefined treatment objectives and resulted in improved hemodynamics and preload sensitivities compared to the CS support.

## Introduction

The prevalence of advanced heart failure (HF), a complex heart syndrome that has long been associated with significant mortality and morbidity rates, has been continuously rising worldwide over the last decades (1–3). For the afflicted patients who account for approximately 1–2% of the general adult population, (3) heart transplantation (HT) is considered the gold standard therapy; however, despite the increased number of heart transplantations performed yearly, the number of recipients continues to significantly outpace the supply of donors (4).

An alternative surgical treatment to mitigate donors' shortage and allow better management of end-stage HF patients is the implantation of ventricular assist devices (VADs) (5, 6). VADs are mechanical pumps that relieve the native heart and restore a fragment of the cardiac output. Their major deployment is complementary, serving as bridge to transplantation or bridge to recovery (7). However, recent studies have shown that VADs are increasingly used as destination therapy, achieving 1- and 2-year survival rates of 82.3 and 73.1%, respectively, which are comparable to HT (8, 9).

Since their initial approval, VADs have been evolved and matured, with the continuous flow turbodynamic VADs (cfVADs) superseding the bulky and failure-prone volume displacement, pulsatile VADs (10, 11). Despite the notably improved reliability and implantability, cfVADs are still associated with right-heart failure, gastrointestinal bleeding, hemorrhagic strokes, and aortic valve insufficiency, which reduce the survival rates and increase hospital readmissions (5, 12–14). These adverse events are often related to the inability of the currently used cfVADs to respond physiologically to the changing perfusion demands of the patients. To ameliorate this, various research groups have been investigating cfVAD control strategies that restore the physiological response of cfVADs (15, 16). The majority of these strategies aim to imitate the Frank-Starling mechanism (17) by adapting the rotational speed of the cfVAD based on feedback provided by hemodynamic parameters that act as preload surrogates (18–21). More complex strategies utilize norm-optimal iterative learning control (22, 23) to regulate the end-diastolic volume. They exploit the repetitive nature of the heart and, hence, use information of previous cycles to deduce the control input for the new cycle. These approaches are promising; however, their performance can be restricted by the accuracy of the cardiovascular system and cfVAD models that are integrated in the control structure to allow the prediction of the control function.

All control strategies mentioned above improve the responsiveness of VADs, however, they do not address the diminished blood pulsatility induced by cfVADs support. Whether or not the diminished pulsatility is among the major contributors of adverse events of cfVADs is still a controversial issue (24). Nonetheless, recent studies have reported strong evidence that the lack of pulsatility can negatively affect the endothelial and peripheral vascular function (25–27) and, hence, increase the risk of non-surgical bleeding (28). Additionally, various studies highlight the better control of ventricular unloading and patient's hemodynamics when VADs that effectively resemble the pulsatile flow conditions are deployed (29–32).

In an attempt to imitate the pulsatile blood pressure and flow waveforms, while exploiting the reliability and implantability of cfVADs, various approaches of cfVAD speed modulation have been proposed in literature (33–38). A recent review shows that predefined speed profiles implemented in synchrony with the native heart can systematically manipulate the ventricular load and the pulsatility in the arterial tree, confirming the positive effect of speed modulation (39). These approaches focus on the modulation of the speed-profile which is readily available in the clinical setting. However, cfVAD speed-profiles are greatly influenced by the VAD design, hindering the deduction of a direct relation to hemodynamics, as well as, their transferability to different VADs. A more intuitive approach is the modulation of cfVAD speed based on predefined pump flow-profiles. By utilizing an iterative learning control (ILC) scheme, Rüschen et al. (40) provided evidence that accurate tracking of optimized pump flow-profiles can be achieved, resulting in a significant reduction of the left ventricular stroke work (LVSW). For the latter study, a detailed model of the VAD is necessary to enable the accurate flow-profile tracking.

In this study, we introduce a physiologic data-driven iterative learning controller (PDD-ILC) for left ventricular cfVADs. The proposed PDD-ILC enables the generation of preload-adaptive reference pump-flow trajectories based on the Frank-Starling mechanism and treatment objectives, such as pulsatility maximization or LVSW minimization, selected by the clinicians. The tracking of the reference flow trajectories is achieved by measuring left ventricular pressure (LVP) and pump flow (PF), and then implementing the data-driven ILC (DD-ILC). The DD-ILC exploits the recurrent nature of the heart cycle to incorporate the errors identified in previous cycles to the control input of the new cycle and, hence, enhance the tracking performance without requiring a system model. Finally, to enable feedback in the time-domain, a proportional-derivative controller is coupled with the PDD-ILC. The performance of the proposed PDD-ILC was assessed with respect to physiologic responsiveness and trajectory tracking with *in-silico* experiments that emulated various physiologic conditions, and compared with a constant speed (CS) controller and a newly developed physiological pump flow proportional-integral-derivative controller (PF-PIDC).

## Methods and Materials

### Cardiovascular System Model

In this work, the performance of the PDD-ILC was assessed solely with *in-silico* experiments, wherein the deployed human cardiovascular system (CVS) was modeled based on the lumped-parameter representation proposed by Colacino et al. (41). In this representation, the systemic and pulmonary circulations were divided into the arterial and venous systems. The arterial and venous systems were modeled with five-element and classic Windkessel models, respectively. The CVS model incorporated autoregulatory mechanisms for the adaptation of the flow resistance in the systemic and pulmonary arterial systems, as well as the adaptation of the unstressed volume in the systemic veins. All four chambers of the heart were included as actively contracting elements and they were defined by a non-linear time-varying elastance model and an energy dissipation term. A detailed description of the model and its validation in physiological and pathological states can be found in the work of Colacino et al. (41). In all simulations, the pathologic conditions defined by Ochsner et al. (42) were used.

### Numerical Models of Blood Pumps

The conditions of a cfVAD supported patient were imitated by coupling the CVS model described above with a numerical model of a non-implantable mixed-flow turbodynamic blood pump (Deltastream DP2, Medos Medizintechnik AG, Stolberg, Germany). The later model was based on the work of Amacher et al. (37). In detail, the mathematical description includes two differential Equations that define the acceleration of fluid (1) and the acceleration of the rotor (2):

(1) $$\begin{array}{r} \frac{dQ}{dt}=\;\frac{1}{L}\left(H\left(Q\left(t\right),\omega \left(t\right)\right)-\left(p_{ds}\left(t\right)-p_{us}\left(t\right)\right)\right) \end{array}$$

(2) $$\begin{array}{r} \frac{d\omega }{dt}=\;\frac{1}{\Theta }\left(-\left(Q\left(t\right),\omega \left(t\right)\right)+k\cdot I\left(t\right)\right)) \end{array}$$

where *Q*, ω, and *I* are the flow-rate, the rotational speed and the current of the pump, respectively. *p*_*ds*_ and *p*_*us*_ are the pressures downstream and upstream of the pump, which correspond to the aortic and left ventricular pressures, respectively. *L* and Θ are the fluid inertance and the rotor inertia, while *k* is the torque-constant of the pump motor. *H* and *T* are matrices containing two-dimensional maps of the pressure across the pump and the hydraulic torque applied on the shaft. The values of the these parameters were retrieved from Amacher et al. (37).

### Overview of PDD-ILC

**Figure 1:**
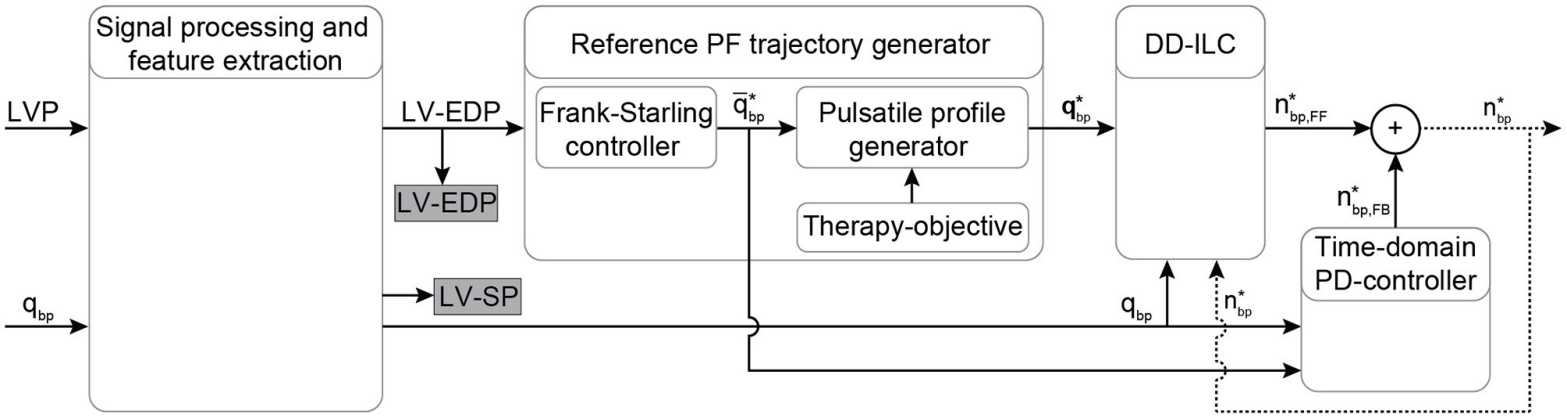
Schematic overview of the physiological data-driven iterative learning controller. The input signals LVP and PF are filtered and the EDP and SP indices are extracted from the LVP. Based on the Frank-starling mechanism, the desired average PF is calculated based on the LV-EDP that is used as a preload surrogate, and the reference pulsatile PF trajectory is obtained based on the objectives of the therapy. A data-driven iterative learning controller is implemented to achieve accurate tracking of the reference PF without requiring modeling of the CVS or the pump. Finally, a proportional-derivative (PD) controller is coupled coupled to enable feedback in the time-domain. The desired pump speed is the output of the controller. Bold letters define vectors. LVP, left ventricular pressure; EDP, end-diastolic pressure; SP, systolic pressure; PF, pump flow; *q*_*bp*_, measured pump flow; ${\bar{q}}_{bp}^{*}$, heart cycle average pump flow; $q_{bp}^{*}$, time vector of the desired pump flow trajectory; $n_{bp,FF}^{*}$, feed forward desired pump speed; $n_{bp,FB}^{*}$, feedback desired pump speed; $n_{bp}^{*}$, desired pump speed output.

The proposed PDD-ILC scheme for LVADs aims to provide an accurate reference tracking of predefined, therapy-oriented, PF profiles while it achieves physiological VAD response when preload changes are encountered. A schematic overview of the PDD-ILC structure is depicted in Figure 1 and it can be divided into four main subsystems, namely, signal processing and feature extraction, reference PF trajectory generator, DD-ILC, and time-domain PD-controller.

#### Signal Processing and Feature Extraction

The function of the PDD-ILC is based on the LVP, specifically the end-diastolic (LV-EDP) value, and the PF. The acquisition of these variables is envisaged by integrating two pressure sensors into a tapered inflow cannula and exploiting the difference in the dynamic pressure component between the measuring ports to estimate the PF, as proposed by von Petersdorff-Campen et al. (43). However, in this *in-silico* study, the simulated signals were used instead and white noise was added in specified experiments to emulate a realistic sensor signal and challenge the PDD-ILC, as described in the section “Experiments for Performance evaluation.” Both LVP and PF signals were low-pass filtered with a first-order filter with cut-off frequency of 25 Hz.

**Figure 2:**
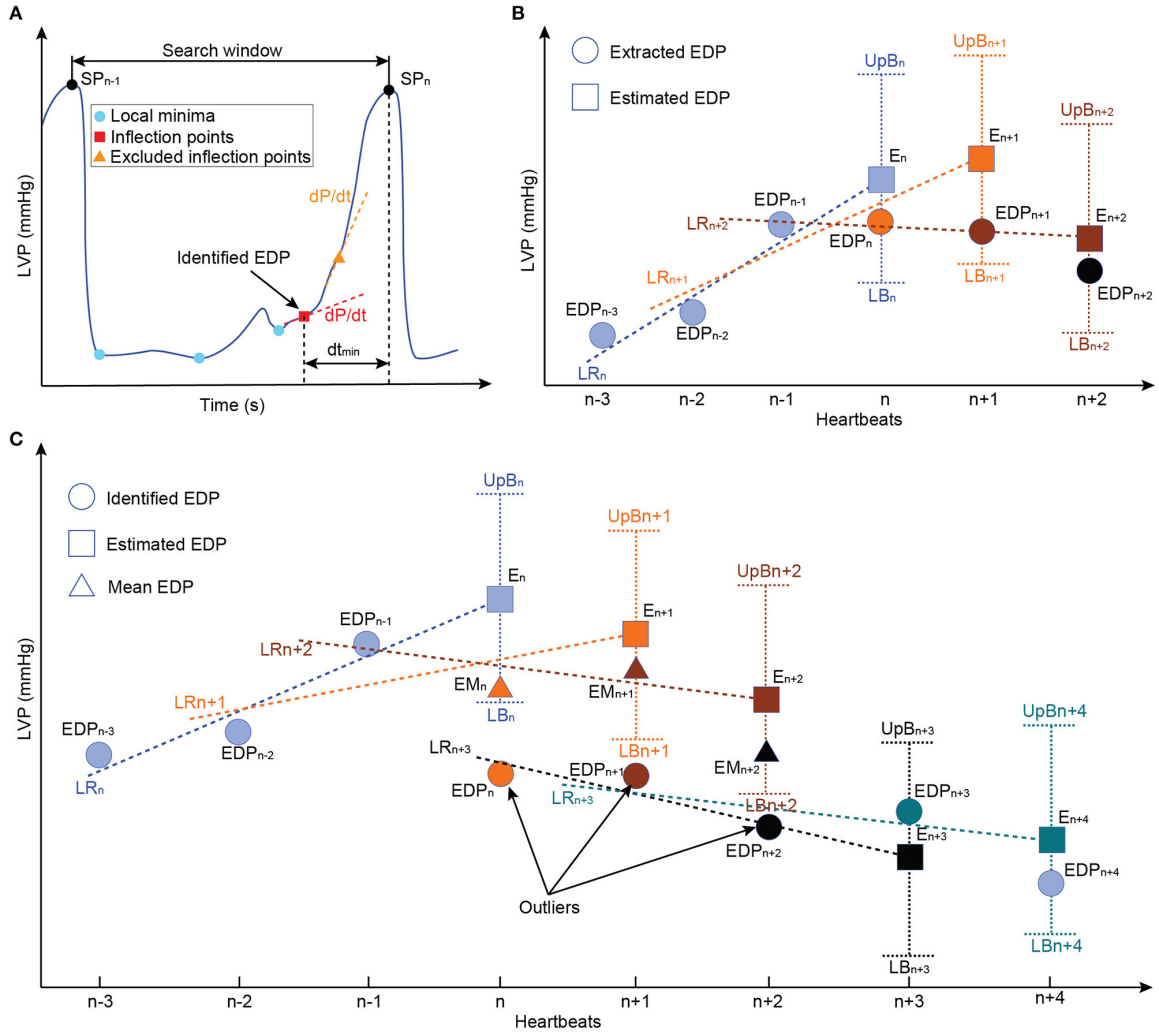
Schematic representation of the LV-EDP extraction process and the extension for the minimization of misdetections. **(A)** Identification of all local minima (cyan circles) and inflection points (red rectangles) as LV-EDP candidates. The inflection points with large first LVP derivative (dLVP/dt > 40 mmHg/s) are excluded and the candidate with the smaller distance from the SP index (dt_min_) is identified as the LV-EDP. **(B)** Comparison of the identified LV-EDP (filled circles) with the LV-EDP estimated (filled rectangles) based on linear regression of the LV-EDP extracted on the previous three heartbeats. If the identified LV-EDP is within the boundaries, it is extracted as LV-EDP value. **(C)** Based on the comparison described in b, if the identified LV-EDP is outside the boundaries it is considered an outlier and the mean value of the last three LV-EDPs is extracted as LV-EDP (filled triangles) of the investigated heartbeat. The outliers are stored in memory and if three consecutive identified LV-EDPs constitute outliners a flag is raised and the new LV-EDP estimate is based on the outliers (LR_n+3_) instead of the extracted LV-EDPs. In this way, physiological rapid changes in preload are not obscured. LVP, left ventricular pressure; EDP, end-diastolic pressure; LR, linear regression; EM, extracted mean value; E, extracted; UpB, upper boundary; LB, lower boundary.

The extraction of the LV-EDP and left ventricular systolic pressure (LV-SP) from the entire time sequence of the LVP was based on the work of Petrou et al. (34). In detail, the LVP was further low-pass filtered with a second-order filter with a cut-off frequency of 8 Hz. From the timeseries data, the indices of the local maxima corresponding to the LV-SP were extracted and the heartbeat was defined as the interval between two consecutive LV-SP indices (Figure 2A). As it is shown in Figure 2A, for each heartbeat, the local minima of the LVP as well as the points of inflection, where the curvature changed from concave to convex, were identified as possible LV-EDP candidates. From the inflection points, only the points where the first derivative of the LVP was below a certain threshold (here 40 mmHg/s) were qualified as possible LV-EDP candidates. From all candidates, the one closest to the LV-SP index was identified as LV-EDP.

Although this approach is accurate, the changes in the LVP waveform due to the in-cycle speed modulation of the cfVAD can increase the LV-EDP misdetections. To address this, an extension for the LV-EDP extraction process was developed in this work. Specifically, as it can be seen in Figure 2, the LV-EDP values identified in the last three heartbeats are used to estimate through linear regression the LV-EDP value of the new heartbeat. The LV-EDP identified for the new heartbeat is compared with the estimate and if it lies within predefined boundaries (here ± 1 mmHg) it is extracted as the LV-EDP. When the identified LV-EDP lies outside the boundaries (Figure 2C), it is considered an outlier and the mean LV-EDP value of the last three heartbeats is extracted as LV-EDP of the new cycle. The latter value is used along with the LV-EDP of the previous two heartbeats for the estimation of the LV-EDP of the next heartbeat. The outlier is saved in memory and if three consecutive outliers have been identified, a flag is created that the LV-EDP has indeed changed significantly and the new LV-EDP estimate is projected from these three outliers (Figure 2C). The latter step is incorporated to ensure that rapid changes in the LV-EDP are not obscured.

#### Reference Pump Flow Trajectory Generator

In this work, reference PF trajectories were used to modulate the pump speed since they provide more intuitive control of the hemodynamics and the interactions between the cfVAD and the CVS, (40, 44) while at the same time, they are highly transferable to different cfVAD designs when a sufficiently accurate tracking performance is guaranteed. The generation of these trajectories followed a two-step approach. As a first step, the Frank-Starling mechanism was imitated to obtain a physiological preload response of the cfVAD. More precisely, the LV-EDP extracted at each heartbeat was used as a surrogate of the preload (*EDP*_*LV*_) and, by assuming that the flow from the aortic valve is negligible and the PF approximates the cardiac output (CO), the linear part of the starling-relationship between the LV-EDP and the CO was used to provide the necessary average PF (${\bar{q}}_{bp}^{*}$) as follows:

(3) $$\begin{array}{r} {\bar{q}}_{bp}^{*}=\;q_{bp,\;ref}+k_{Fs}\left(EDP_{LV}-EDP_{LV,ref}\right) \end{array}$$

Here *q*_*bp,ref*_ and *EDP*_*LV,ref*_ are reference values for the PF and LV-EDP, respectively, identified from the healthy heart at rest conditions and a CO of 5 *L*/*min* during calibration. The parameter *k*_*Fs*_ denotes the preload sensitivity which can be selected by the clinician. This ability to directly select the preload sensitivity is paramount to achieve a patient-specific response of the controller and constitutes a major advantage compared to speed-based controllers, where the control gain needs to be tuned to reach a satisfying preload sensitivity.

**Figure 3:**
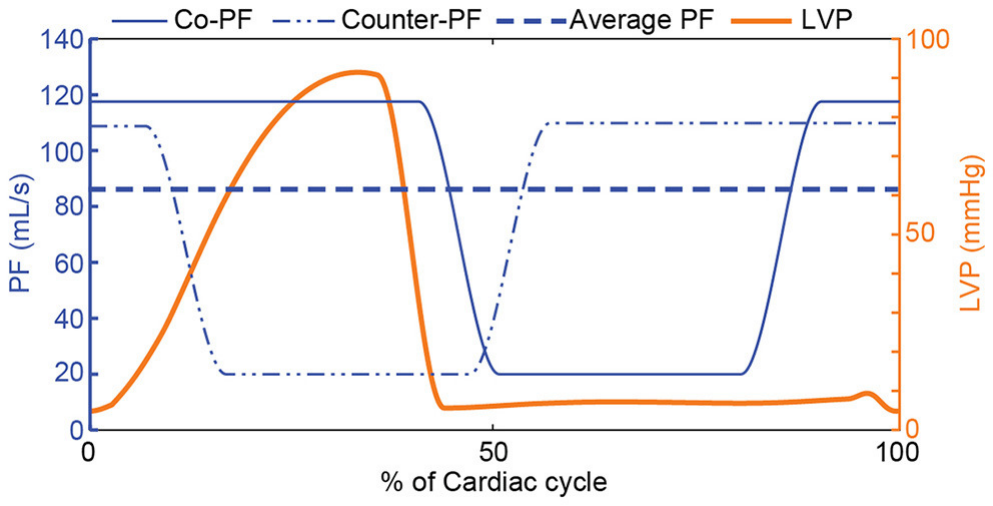
Reference PF trajectories that incorporate a physiological response to preload changes based on the Frank-Starling mechanism and therapy-oriented pulsatile PF profiles. A copulsation and a counterpulsation PF trajectory along with the LVP for one cardiac cycle are depicted. For both PF trajectories, the minimum PF is $q_{bp,min}^{*}=20\;mL/s$ and the maximum PF is calculated based on the $q_{bp,min}^{*}$ and the necessary average PF provided by the starling-relation (${\bar{q}}_{bp}^{*}$). PF, pump flow; LVP, left ventricular pressure; Co-PF, copulsation pump flow; Counter-PF, counterpulsation pump flow.

The second step to obtain the reference PF trajectories was to incorporate an in-cycle pulsatile profile that enables the accomplishment of treatment-based objectives selected by the clinician. These pulsatile trajectories were based on trapezoidal profiles, wherein the minimum PF was selected to be $q_{bp,min}^{*}=20\;mL/s$ to provide a safety margin against backflow, and the maximum PF was calculated based on the $q_{bp,min}^{*}$ and the necessary average PF provided by the starling-relation (${\bar{q}}_{bp}^{*}$). For each trajectory, the minimum flow was applied for 30% of the cardiac cycle, the maximum flow for 50% of the cardiac cycle, while each transition phase had a duration of 10% of the cardiac cycle. This proportion was chosen to prevent short spikes of desired maximum flow, since such trajectories could not be tracked using cfVADs and would be susceptible to high blood damage. For the pulsatile trajectories, various phase shifts (45) with respect to the onset of cardiac cycle can be applied to achieve different concurrent objectives; however, in this work, only copulsation and counterpulsation trajectories were investigated to achieve maximization of the aortic pulse pressure and minimization of LVSW, respectively, as proposed by Ising et al. (44). The maximum PF was applied during the entire systole for the copulsation trajectory, whereas for the counterpulsation trajectory the maximum PF was applied during diastole. In Figure 3 the reference PF trajectories for an average PF of 85 mL/s are depicted.

#### Data-Driven Iterative Learning Controller

To achieve the desired response of the PDD-ILC, accurate reference tracking of the PF trajectory is required. Considering the repeating disturbances applied on the cfVAD in each cardiac cycle by the changes in the head pressure (difference between pressure at the outlet and pressure at the inlet of the cfVAD) from the remaining heart function, as well as the periodic changes in the preload, ILC schemes are suitable for reference trajectory tracking. More precisely, in repetitive process, ILC strategies can exploit the knowledge obtained in previous iterations to produce a feed-forward control input that progressively enhances the tracking performance. In this study, the DD-ILC was developed based on the approach proposed by Chi et al. (46) wherein a pseudo partial derivative (PPD) computed from the input and output signals serves as system model in the iteration domain, where one iteration stands for one heartbeat. The model is then used in a quadratic optimization procedure to minimize a cost function subject to input and output constraints.

**Figure 4:**
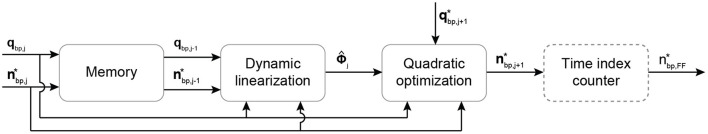
Schematic overview of the DD-ILC algorithm. The pump speed setpoint and the PF are stored in a memory block. Then, they are used at the beginning of each cycle to update the system model through dynamic linearization. The model is used in a quadratic optimization problem to minimize the PF tracking error under pump speed constraints. The time index counter operates continuously to extract and output the feedforward pump speed setpoint at every time step within an iteration. Bold letters define vectors. $n_{bp,j}^{*}$, pump speed setpoint vector of the *j*^*th*^ cycle; ***q***_*bp,j*_, measured PF vector of the *j*^*th*^ *cycle;* ${\hat{\Phi }}_{j}$, pseudo partial derivative denoting the linearized system model; $q_{bp,j+1}^{*}$, PF reference trajectory; $n_{bp,FF}^{*}$, feedforward pump speed setpoint in the time domain.


The implementation of the DD-ILC is illustrated in Figure 4. Initially, a memory block is incorporated to store the per-cycle vectors of the pump speed setpoint $n_{bp}^{*}$ and the PF ***q***_*bp*_, with varying number of samples *N*:

(4) $$\begin{array}{r} n_{bp,j}^{*}=\left[n_{bp,j}^{*}\left(0\right),n_{bp,j}^{*}\left(1\right),\ldots ,n_{bp,j}^{*}\left(N-1\right),n_{bp,j}^{*}\left(N\right)\right] \end{array}$$

(5) $$\begin{array}{r} q_{bp,j}=\left[q_{bp,j}\left(0\right),q_{bp,j}\left(1\right),\ldots ,q_{bp,j}\left(N-1\right),q_{bp,j}\left(N\right)\right] \end{array}$$

These vectors contain the information of the entire time sequences of the previous cycles (iterations). They are used to obtain a representation of the CVS and the cfVAD system in the iteration domain through dynamic linearization. Specifically, the dynamic linearization model is based on the identification of the PPD ***Φ***_*j*_ by relating the difference in the output signal ***q***_*bp*_ and the input signal $n_{bp}^{*}$ between consecutive iterations:

(6) $$\begin{array}{r} \Delta q_{bp,j}=\Phi_{j}\Delta n_{bp,j}^{*}\\ with\;\Delta q_{bp,j}=q_{bp,j}-q_{bp,j-1}\;,\;\Delta n_{bp,j}^{*}=n_{bp,j}^{*}-n_{bp,j-1}^{*} \end{array}$$

where *j* denotes the iteration index. Since the system is causal, ***Φ***_*j*_ is a lower triangular matrix. To compute an estimate of the PPD, denoted as ${\hat{\Phi }}_{j}$, the update formula described by Chi et al. (46) was used:

(7) $$\begin{array}{r} {\hat{\varphi }}_{j+1}^{t}={\hat{\varphi }}_{j}^{t}+\frac{\eta \left(\Delta q_{bp,j}\left(k+1\right)-{\hat{\varphi }}_{j}^{t}{\Delta n}_{bp,j}^{*}\left(k\right)\right){\Delta n}_{bp,j}^{*T}\left(k\right)}{\mu +||{\Delta n}_{bp,j}^{*}\left(k\right)|{|}^{2}} \end{array}$$

where ${\hat{\varphi }}_{j+1}^{t}$ denotes the nonzero vector of the (*t* + 1)^*th*^ row of ${\hat{\Phi }}_{j+1}$. Accordingly, $n_{bp,j}^{*}\left(k\right)$ contains the PF setpoints at iteration *j* up to time index *k* and based on Equation (4) is a varying dimension vector with *k* elements. The learning process can be tuned by selecting the normalization value μ and the learning gain η. The values of the later parameters were identified through the controller gain optimization described in section “Optimization of Controller Parameters”.

For the first iteration, the initial values for the pump speed setpoint, the PF and the PPD required from the algorithm were selected as:


(8) $${\hat{\Phi }}_{0}=10^{-4}\left(\begin{array}{c} \begin{array}{c} 1\;0\\ 1\;1 \end{array}\;\begin{array}{c} \ldots \;0\\ \ldots \;0 \end{array}\\ \begin{array}{c} \vdots \;\ddots \\ 1\;1 \end{array}\;\begin{array}{c} \ddots \;0\\ \ldots \;1 \end{array} \end{array}\right),\;n_{bp,0}^{*}=\left(\begin{array}{c} \begin{array}{c} 0\\ 0 \end{array}\\ \begin{array}{c} \vdots \\ 0 \end{array} \end{array}\right),\;q_{bp,0}^{*}=\left(\begin{array}{c} \begin{array}{c} 0\\ 0 \end{array}\\ \begin{array}{c} \vdots \\ 0 \end{array} \end{array}\right)$$

Hence, no model knowledge is required to initialize the controller. None of the previously converged solutions were used in the initialization procedure, and no model knowledge was included.

As a next step, the estimated PPD is used in a quadratic optimization problem to minimize the predicted PF tracking error under actuator constraints. The cost function in this optimization problem comprises two terms, namely the predicted PF tracking error (*J*_*q*_) and the change in the input vector (*J*_*u*_). The *J*_*u*_ cost component provides robustness against undesirably high changes in the pump speed setpoint during the transient behavior of the learning algorithm. The predicted PF tracking error to be minimized is described as:

(9) $$\begin{array}{r} e_{j}=q_{bp,j+1}^{*}-\;{\hat{q}}_{bp,j+1}^{*} \end{array}$$

where ${\hat{q}}_{bp,j+1}^{*}$ denotes the predicted PF at cycle *j* + 1 using the updated PPD given by:

(10) $$\begin{array}{r} {\hat{q}}_{bp,j+1}^{*}={\hat{q}}_{bp,j}^{*}+{\hat{\Phi }}_{j+1}\;\Delta n_{bp,j}^{*} \end{array}$$

Hence, by combining the two cost components, the cost function can be written as:

(11) $$\begin{array}{r} J_{j+1}=J_{q,j+1}+J_{u,j+1}=e_{j}^{T}Q\;e_{j}+{\Delta n}_{bp,j+1}^{*T}R{\Delta n}_{bp,j+1}^{*} \end{array}$$

where ***Q*** and ***R*** are positive definite weighting matrices that, in this work, are identified during the controller gain optimization described in section “Optimization of Controller Parameters”.

Additionally, to avoid unrealistic pump speed setpoints, the pump speed is constrained between a minimum and maximum value defined based on the pump design. Hence, the final optimization problem can be written as:

(12) $$\begin{array}{r} min_{\Delta n_{bp,j+1}^{*}}(J_{j+1}) \end{array}$$

(13) $$\begin{array}{r} s.\;t.\;\Delta n_{bp,j+1}^{*}\geq n_{bp,min}-n_{bp,j}^{*} \end{array}$$

(14) $$\begin{array}{r} \;\Delta n_{bp,j+1}^{*}\leq n_{bp,max}-n_{bp,j}^{*} \end{array}$$

The optimization problem is solved using the quadprog function provided by MATLAB R2020b (The MathWorks Inc., Natick, MA, USA). The optimized change in the pump speed setpoint vector is added to the speed setpoint vector of the previous iteration to provide the new control input vector as:

(15) $$\begin{array}{r} n_{bp,j+1}=n_{bp,j}+\Delta n_{bp,j+1}^{*} \end{array}$$

Finally, since the dynamic linearization and the quadratic optimization are executed only at the beginning of each cycle, an additional module that operates at the full control frequency extracts the feedforward pump speed setpoint $n_{bp,FF}^{*}$ at every time index.

#### Time-Domain Proportional-Derivative Controller

The DD-ILC incorporates feedback in the iteration domain; however, it is a feedforward controller in the time domain. Therefore, an additional PD controller that operates in parallel to the DD-ILC is introduced (Figure 1). The PD controller showed to deteriorate the convergence speed of the DD-ILC during transient phases. However, it restricts the tracking error to become unbounded when the desired average PF changes rapidly due to changes in LV EDP. Hence, to exploit the latter characteristic without compromising convergence, the time domain PD controller is only activated if the desired average PF changes by at least 1 *mL*/*s* and, therefore, it is described as:


(16) $$\begin{array}{l} n_{bp,FB}^{*}(k)=\;\\ \;\{\begin{array}{c} k_{p}\left(q_{bp}^{*}\left(k\right)-q_{bp}(k)\right)+k_{d}\frac{d\left(q_{bp}^{*}\left(k\right)-q_{bp}(k)\right)}{dk}\;if\;\left|{\bar{q}}_{bp,j+1}^{*}-{\bar{q}}_{bp,j}^{*}\right|\geq 1\\ \;\begin{array}{c} 0\;Otherwise \end{array} \end{array} \end{array}$$

Finally, by incorporating the feedback in the iteration domain provided by the DD-ILC and the feedback in the time domain provided by the PD controller, the pump speed setpoint at time index *k* is given by:

(17) $$\begin{array}{r} n_{bp}^{*}\left(k\right)=n_{bp,FF}^{*}\left(k\right)+n_{bp,FB}^{*}\left(k\right) \end{array}$$

### Physiological Flow PID Controller

**Figure 5:**
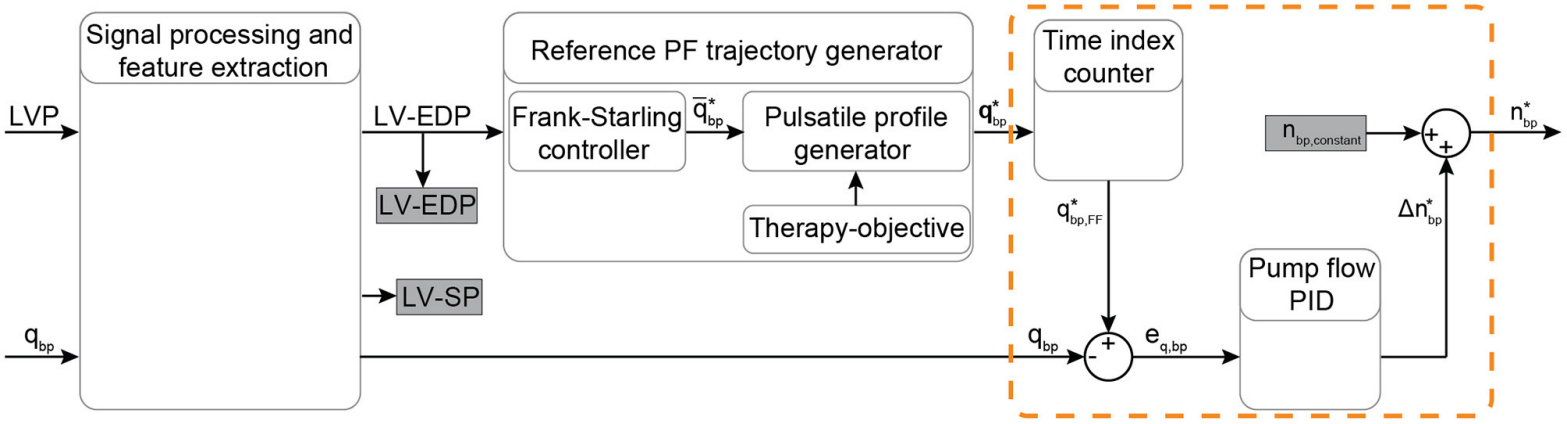
Schematic overview of the physiological flow PID controller. The input signals LVP and PF are filtered and the EDP and SP indices are extracted from the LVP. Based on the Frank-Starling mechanism, the average PF is calculated based on the LV-EDP that is used as a preload surrogate, and the reference pulsatile PF trajectory is obtained based on the objectives of the therapy. A time index counter operates continuously to extract and output the feedforward PF setpoint at every time step. A PID flow controller (included in the orange dashed-line box) provides the necessary change in the pump speed setpoint based on the error between the measured and the desired PF. The desired pump speed is the output of the controller. Bold letters define vectors. LVP, left ventricular pressure; EDP, end-diastolic pressure; SP, systolic pressure; PF, pump flow; PID, proportional-integral-derivative; *q*_*bp*_, measured pump flow; ${\bar{q}}_{bp}^{*}$, heart cycle average pump flow; $q_{bp}^{*}$, time vector of the desired pump flow trajectory; $q_{bp,FF}^{*}$, feed forward desired pump flow; *e*_*q, bp*_, feedback error between the measured and the desired PF; ${\Delta n}_{bp}^{*}$, desired pump speed change; *n*_*bp,constant*_, pump speed constant; $n_{bp}^{*}$, desired pump speed output.

A PID controller was also developed to achieve PF tracking and it was used to further evaluate the performance of the PDD-ILC. This controller uses the LVP and the PF to regulate the pump speed and achieve a physiological response to preload changes while it tracks specific PF profiles. The signal processing and feature extraction blocks, as well as the flow trajectory generator are the same as described for the PDD-ILC. As it can been seen in Figure 5, a time index counter is used after the flow trajectory generator to extract the feedforward PF setpoint at every time step. The measured PF is compared with the PF setpoint and the error is used as feedback to the PID controller. The output of the PID controller corresponds to the desired change in the pump speed. This change is added to a pump speed constant and the desired pump speed is defined. The pump speed is not constrained between a minimum and maximum value as in the PDD-ILC, however, the step-change in pump speed is constraint to 2,500 rotations per minute.

### Experiments for Performance Evaluation

The assessment of the PDD-ILC was based on *in-silico* experiments that simulate a pathologic CVS supported by a cfVAD, using the numerical models described in sections “Cardiovascular System Model” and “Numerical Models of Blood Pumps”. Additionally, to allow a detailed evaluation, several clinical conditions and everyday scenarios emulating resting (Exp0), preload variations (Exp1), afterload variations (Exp2), sleep-to-wake (Exp3), contractility variations (Exp4) as well as rest-to-exercise (Exp5), were tested with the PDD-ILC regulating the cfVAD speed. The parameters of the CVS, as well as the specific values used to simulate the aforementioned conditions are based on the experimental procedure described by Petrou et al. (19) and they are given in Supplementary Tables S1–S3 of the supplementary material. To test the robustness of the PDD-ILC when real measured signals are used instead of the simulated ones, all experiments were repeated with white noise with a variance of 0.86 mmHg^2^ (Exp0n−5n) and 1.72 mmHg^2^ (Exp0nn−5nn) on the LVP or/and a variance of 0.86 (mL/s)^2^ (Exp0n−5n) and 1.72 (mL/s)^2^ (Exp0nn−5nn) PF signals.

To benchmark the performance of the proposed PDD-ILC in comparison to the state of the art, the same experiments have been conducted with a simulated healthy heart (HH), wherein the contractility parameter was set to 1, a constant speed controller (CS), and the developed PF-PIDC. All experiments were executed on MATLAB/Simulink R2020b (The MathWorks Inc., Natick, MA, USA) for 200 s.

### Optimization of Controller Parameters

The performance of the PDD-ILC, as well as the PF-PIDC, is highly dependent on the selection of the control parameters. Although for PID controllers the Ziegler-Nichols approach (47) is most commonly used to fine-tune their parameters (*K*_*P*_, *K*_*I*_, *K*_*D*_), its applicability to non-linear, time-variant systems, such as the CVS system, is prohibited. For the developed PDD-ILC, which includes six control parameters (μ, η, *Q, R, k*_*p*_, *k*_*d*_), there is no intuitive method to fine-tune these parameters.

In this work, the genetic algorithm-based optimization framework (GAOF) described by Magkoutas et al. (48) was used to obtain a set of optimal parameters for each controller. In this framework, the user defines the VAD control structure, the numerical model of the CVS coupled with the numerical model of the selected VAD, the objective function (OF) to be evaluated, the experiments for the evaluation of the OF, and the genetic algorithm (GA) parameters. During the execution, each individual, defined as a set of control parameters, is fed to the controller and the numerical model of the VAD-supported heart is simulated. The simulation results are used for the evaluation of the OF and the obtained value is assigned to the respective individual as “score.” As long as the convergence criterion of the optimization problem is not met and the maximum number of generations (each generation includes multiple individuals) is not achieved, the scores of the individuals are fed to the genetic algorithm. Based on those scores, the GA uses genetic operations, namely elitism, crossover, and mutation, to create new individuals for the next generation. The process continues for each individual and each generation until an optimum (or multiple) set of control parameters has been identified.

To enable the execution of the GAOF for the PDD-ILC and the PF-PIDC, the numerical model of the CVS and the cfVAD described in sections “Cardiovascular System Model” and “Numerical Models of Blood Pumps” were used. The contractility parameter of the CVS was set to 34% of the value described for the healthy heart, emulating a pathological circulation. The experiments Exp1–Exp5 described in section “Experiments for Performance Evaluation” were used for the evaluation of the OF.

For each controller, a two-objective optimization problem was defined, aiming to minimize the overall error in tracking the reference PF trajectory. For the first objective, the root-mean-square-error (RMSE) of the tracking error was initially calculated for each cardiac cycle by:

(18) $$\begin{array}{r} RMSE_{j}=\sqrt{\frac{\sum_{k=1}^{N}{\left(q_{bp,j}\left(k\right)-\;q_{bp,j}^{*}\left(k\right)\right)}^{2}}{N}} \end{array}$$

where *j* denotes the index of the cardiac cycle, *k* denotes the time index and *N* the total number of time indices within the cardiac cycle *j*. As a next step, to ensure that only converged cycles are considered, the last 80 cycles of each experiment (*m*) were obtained and the mean value of *RMSE* was calculated as:

(19) $$\begin{array}{r} {\bar{RMSE}}_{m}=\sqrt{\frac{\sum_{j=0}^{80}RMSE_{j}}{80}} \end{array}$$

Hence, the first objective function including the mean value of the *RMSE* for the six experiments was defined as:

(20) $$\begin{array}{l} J_{1}=a_{1}{\bar{RMSE}}_{1}+a_{2}{\bar{RMSE}}_{2}+a_{3}{\bar{RMSE}}_{3}+a_{4}{\bar{RMSE}}_{4}+\\ \;a_{5}{\bar{RMSE}}_{5}+a_{6}{\bar{RMSE}}_{6} \end{array}$$

where *a*_1_ = 0.2, *a*_2_ = 0.2, *a*_3_ = 0.15, *a*_4_ = 0.15, *a*_5_ = 0.15, and *a*_6_ = 0.15 are weighting factors corresponding to experiments Exp1–6. The latter factors allow the experiments that account for a major fraction of the everyday life of a patient to have a greater influence on the value of the OF.

The second objective of the optimization problem was developed to evaluate the individuals regarding the convergence of the tracking error. Hence, the standard deviation of the *RMSE* in the last 80 cycles of each experiment (*m*) was calculated as:

(21) $$\begin{array}{r} {\bar{std}}_{m}=\sqrt{\frac{\sum_{j=1}^{80}{\left(RMSE_{j}-{\bar{RMSE}}_{m}\right)}^{2}}{80}} \end{array}$$

Accounting the terms of all experiments and using the weighting factors described for J_1_, the second objective function is defined as:

(22) $$\begin{array}{r} J_{2}=a_{1}{\bar{std}}_{1}+a_{2}{\bar{std}}_{2}+a_{3}{\bar{std}}_{3}+a_{4}{\bar{std}}_{4}+a_{5}{\bar{std}}_{5}+a_{6}{\bar{std}}_{6} \end{array}$$

To avoid unrealistic control parameters, their values were constrained between a minimum and a maximum value given in Supplementary Table S4 in the Supplementary Material. Hence, the final optimization problem for the PDD-ILC was described as:

(23) $$\begin{array}{r} min_{x}\left(\;J_{1}\left(x\right),J_{2}\left(x\right)\right) \end{array}$$

(24) $$\begin{array}{r} s.\;t.\;\mu_{min}\leq \mu \geq \mu_{max} \end{array}$$

(25) $$\begin{array}{r} \;\eta_{min}\leq \eta \geq \eta_{max} \end{array}$$

(26) $$\begin{array}{r} \;Q_{min}\leq Q\geq Q_{max} \end{array}$$

(27) $$\begin{array}{r} \;R_{min}\leq R\geq R_{max} \end{array}$$

(28) $$\begin{array}{r} \;k_{p,min}\leq k_{p}\geq k_{p,max} \end{array}$$

(29) $$\begin{array}{r} \;k_{d,min}\leq k_{d}\geq k_{d,max} \end{array}$$

where ***x*** denotes the set of control parameters (μ, η, *Q, R, k*_*p*_, *k*_*d*_). The optimization problem for the PF-PIDC, wherein the set of control parameters was **x** = (*K*_*P*_, *K*_*I*_, *K*_*D*_), was described as:

(30) $$\begin{array}{r} min_{x}\left(\;J_{1}\left(x\right),J_{2}\left(x\right)\right) \end{array}$$

(31) $$\begin{array}{r} s.\;t.\;K_{P,min}\leq K_{P}\geq K_{P,max} \end{array}$$

(32) $$\begin{array}{r} \;K_{I,min}\leq K_{I}\geq K_{I,max} \end{array}$$

(33) $$\begin{array}{r} \;K_{D,min}\leq K_{D}\geq K_{D,max} \end{array}$$

where the minimum and maximum constraint values are given in Supplementary Table S4 in the Supplementary Material.

The solution of the described two-objective optimization problem did not provide a single optimum solution, but a set of non-dominated solutions (pareto front) that were chosen as optimal if any of the objectives could not be improved without sacrificing the other objective. Hence, from the several individuals included in the pareto front of each controller, the final control parameters (Table 1) were selected after evaluating the overall performance of several sets of optimum parameters under the dynamic tests described in section “Experiments for Performance Evaluation”.

**Table 1:** Optimized control parameters for PDD-ILC and flow PID controllers.

**PDD-ILC**		**Flow PID**	
μ	0.7315	*K*_*P*_	401.23
η	0.7859	*K*_*I*_	67.51
*Q*	120.7388	*K*_*D*_	19.15
*R*	0.1365		
*k*_*p*_	3.2155		
*k*_*d*_	3.1926		

For both controllers, the optimization problem was solved by using the multi-objective genetic algorithm provided in the global optimization toolbox of MATLAB. The default parameters of the genetic algorithm were applied for the genetic operations, while the initial population and the size of each generation was 500 individuals. The convergence criteria were met when for 15 consecutive generations any new individual was included in the pareto front, or when a maximum number of 50 generations was reached.

## Results

### Trajectory Tracking and Convergence

**Figure 6:**
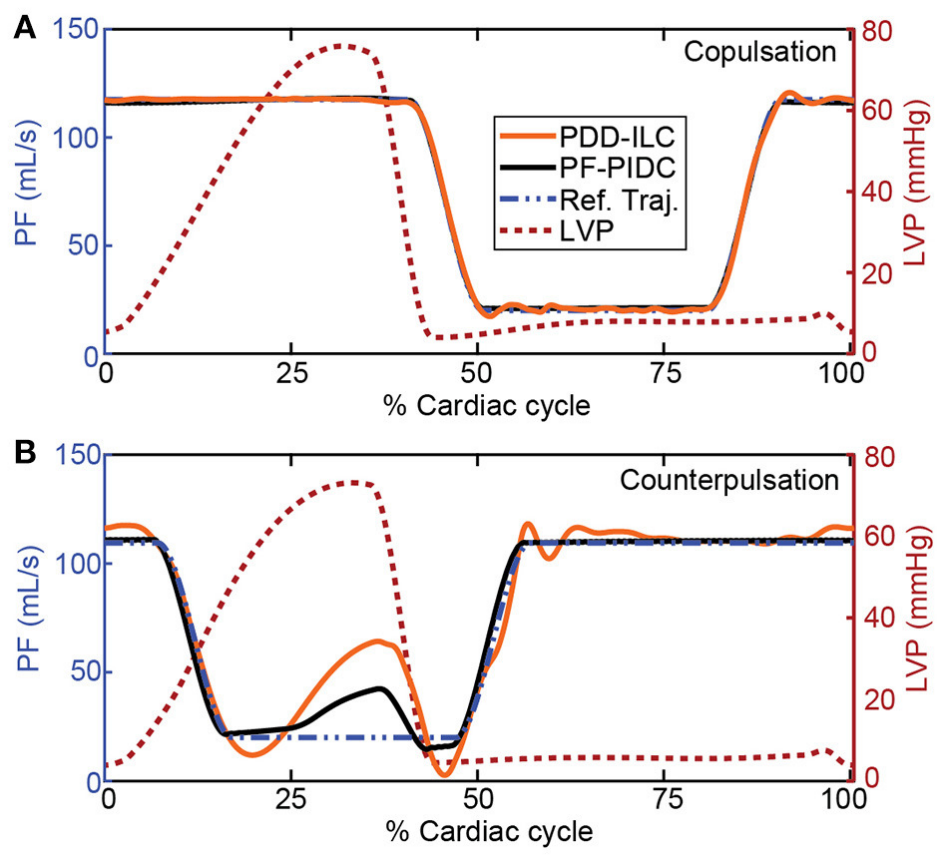
Reference trajectory tracking performance of the PDD-ILC and the PF-PIDC during one cardiac cycle of the rest-conditions experiment (Exp0, Supplementary Table S1, Supplementary Material) under **(A)** copulsation and **(B)** counterpulsation. The reference trajectories, along with the left ventricular pressure profile that corresponds to the main source of disturbance are given for both modes. Both controllers are able to track accurately the reference trajectory during copulsation, reaching the maximum and minimum PF values without time lag. During the counterpulsation, the high disturbance of the fast change in LVP cannot be compensated completely from any of the controllers, however, the overall tracking is adequate. PDD-ILC, physiologic data-driven iterative learning controller; PF-PIDC, pump flow proportional-integral-derivative controller; Ref. Traj., reference trajectory; LVP, left ventricular pressure.

**Figure 7:**
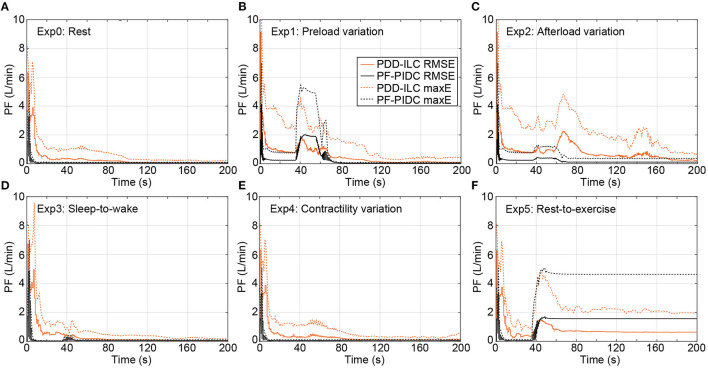
Transient performance of the PDD-ILC and the PF-PIDC in terms of RMSE and maximum instantaneous error in tracking the reference trajectory under all physiological conditions and scenarios executed with the copulsation mode. **(A)** Rest-conditions (Exp0): The PDD-ILC converged after 100 iterations, obtaining an RMSE of $0.07\;L\;\overset{-1}{\min}$ and maximum error of $0.19\;L\;\overset{-1}{\min}$. The PF-PIDC obtained an RMSE of $0.06\;L\;\overset{-1}{\min}$ and maximum error of $0.11\;L\;\overset{-1}{\min}$. **(B)** Preload variation (Exp1): Last variation at 85 seconds. The converged RMSE and maximum error for the PDD-ILC was $0.07\;L\;\overset{-1}{\min}$ and $0.23\;L\;\overset{-1}{\min}$, respectively. The RMSE and maximum error for the PF-PIDC was $0.05\;L\;\overset{-1}{\min}$ and $0.08\;L\;\overset{-1}{\min}$, respectively. **(C)** Afterload variation (Exp2): Last variation at 85 seconds. The converged RMSE and maximum error for the PDD-ILC was $0.20\;L\;\overset{-1}{\min}$ and $0.73\;L\;\overset{-1}{\min}$, respectively. The RMSE and maximum error for the PF-PIDC was $0.11\;L\;\overset{-1}{\min}$ and $0.38\;L\;\overset{-1}{\min}$, respectively. **(D)** Sleep-to-wake (Exp3): Last variation at 85 seconds. The converged RMSE and maximum error for the PDD-ILC was $0.05\;L\;\overset{-1}{\min}$ and $0.21\;L\;\overset{-1}{\min}$, respectively. The RMSE and maximum error for the PF-PIDC was $0.06\;L\;\overset{-1}{\min}$ and $0.11\;L\;\overset{-1}{\min}$, respectively. **(E)** Contractility variation (Exp4): Last variation at 85 seconds. The converged RMSE and maximum error for the PDD-ILC was $0.05\;L\;\overset{-1}{\min}$ and $0.18\;L\;\overset{-1}{\min}$, respectively. The RMSE and maximum error for the PF-PIDC was $0.06\;L\;\overset{-1}{\min}$ and $0.10\;L\;\overset{-1}{\min}$, respectively. **(F)** Rest-to-exercise (Exp5): Last variation at 85 seconds. The converged RMSE and maximum error for the PDD-ILC was $0.68\;L\;\overset{-1}{\min}$ and $2.46\;L\;\overset{-1}{\min}$, respectively. The RMSE and maximum error for the PF-PIDC was $1.56\;L\;\overset{-1}{\min}$ and $4.63\;L\;\overset{-1}{\min}$, respectively. RMSE, root mean square error; maxE, maximum error; PDD-ILC, physiologic data-driven iterative learning controller; PF-PIDC, pump flow proportional-integral-derivative controller.

**Figure 8:**
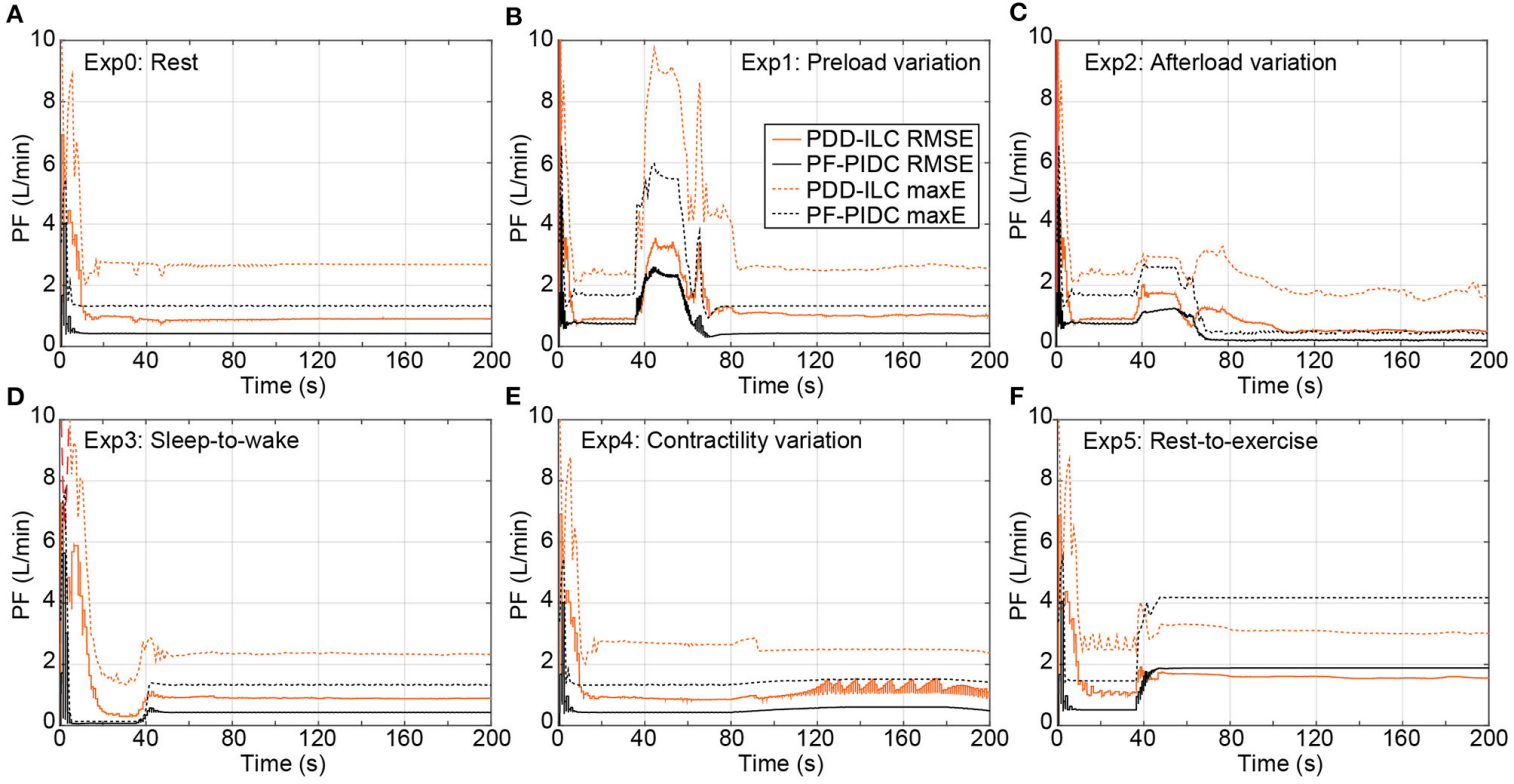
Transient performance of the PDD-ILC and the PF-PIDC in terms of RMSE and maximum instantaneous error in tracking the reference trajectory under all physiological conditions and scenarios executed with the counterpulsation mode selected. **(A)** Rest-conditions (Exp0): The PDD-ILC converged after 50 iterations, obtaining an RMSE of $0.88\;L\;\overset{-1}{\min}$ and maximum error of $2.64\;L\;\overset{-1}{\min}$. The PF-PIDC obtained an RMSE of $0.42\;L\;\overset{-1}{\min}$ and maximum error of $1.32\;L\;\overset{-1}{\min}$. **(B)** Preload variation (Exp1): Last variation at 85 seconds. The converged RMSE and maximum error for the PDD-ILC was $0.94\;L\;\overset{-1}{\min}$ and $2.60\;L\;\overset{-1}{\min}$, respectively. The RMSE and maximum error for the PF-PIDC was $0.43\;L\;\overset{-1}{\min}$ and $1.32\;L\;\overset{-1}{\min}$, respectively. **(C)** Afterload variation (Exp2): Last variation at 85 seconds. The converged RMSE and maximum error for the PDD-ILC was $0.50\;L\;\overset{-1}{\min}$ and $1.67\;L\;\overset{-1}{\min}$, respectively. The RMSE and maximum error for the PF-PIDC was $0.19\;L\;\overset{-1}{\min}$ and $0.50\;L\;\overset{-1}{\min}$, respectively. **(D)** Sleep-to-wake (Exp3): Last variation at 85 seconds. The converged RMSE and maximum error for the PDD-ILC was $0.90\;L\;\overset{-1}{\min}$ and $2.30\;L\;\overset{-1}{\min}$, respectively. The RMSE and maximum error for the PF-PIDC was $0.43\;L\;\overset{-1}{\min}$ and $1.34\;L\;\overset{-1}{\min}$, respectively. **(E)** Contractility variation (Exp4): Last variation at 85 seconds. The converged RMSE and maximum error for the PDD-ILC was $1.08\;L\;\overset{-1}{\min}$ and $2.36\;L\;\overset{-1}{\min}$, respectively. The RMSE and maximum error for the PF-PIDC was $0.48\;L\;\overset{-1}{\min}$ and $1.41\;L\;\overset{-1}{\min}$, respectively. **(F)** Rest-to-exercise (Exp5): Last variation at 85 seconds. The converged RMSE and maximum error for the PDD-ILC was $1.61\;L\;\overset{-1}{\min}$ and $3.06\;L\;\overset{-1}{\min}$, respectively. The RMSE and maximum error for the PF-PIDC was $1.88\;L\;\overset{-1}{\min}$ and $4.18\;L\;\overset{-1}{\min}$, respectively. RMSE, root mean square error; maxE, maximum error; PDD-ILC, physiologic data-driven iterative learning controller; PF-PIDC, pump flow proportional-integral-derivative controller.

The performance of the PDD-ILC and the PF-PIDC in tracking the PF reference trajectories was evaluated under all physiological conditions simulated with the experiments described in section “Experiments for Performance Evaluation” for copulsation and counterpulsation modes (Figures 6–**8**). In Figure 6, the tracking performance during rest conditions (Exp0, Supplementary Table S1, Supplementary Material) is shown for one cardiac cycle with both controllers being converged. When the copulsation mode is selected (Figure 6A), both controllers show excellent performance with the minimum and maximum PF values being achieved without overshoot and time delay. During the counterpulsation mode the tracking is accurate and without time delays in the transition phases (Figure 6B). However, the highly changing pressure conditions applied on the cfVAD during the contraction of the LV deteriorate the tracking performance in the region of low PF of both controllers.

In Figure 7, the RMSE calculated based on Equation (18) as well as the maximum instantaneous tracking error computed for each cardiac cycle are depicted for all physiological experiments (Ex0–5, Supplementary Table S1, Supplementary Material) under copulsation mode. During the rest-conditions experiment (Exp0), the PDD-ILC obtained an RMSE below 0.33 *L min*^−1^ after 30 iterations and converged to 0.07 *L min*^−1^ after 100 iterations (Figure 7A). Except for the initial 10 iterations, wherein the system was not settled, the maximum tracking error remained below 1.21 *L min*^−1^ and reduced continuously to achieve 0.19 *L min*^−1^ after convergence. During the same experimental conditions, the PF-PIDC obtained an RMSE of 0.06 *L min*^−1^ and a maximum error of 0.11 *L min*^−1^. During the preload variations (Exp1), the controllers showed an increase in both the RMSE and the maximum error during the transition phases of the experiment, however after the last transition (at about 75 s) both reached the error values achieved in Exp0, with the PDD-ILC converging in <60 iterations. As depicted in Figure 7C, the PF-PIDC showed a slightly increased RMSE of 0.24 *L min*^−1^ during the afterload experiment (Exp2). In this setting, the PDD-ILC also presented higher RMSE and maximum error throughout the entire experiment, achieving an RMSE of 0.87 *L min*^−1^ at the end of the experiment. During the sleep-to-wake (Exp3) and contractility variation (Exp4) settings both controllers showed excellent tracking performance, resulting in RMSE and maximum error values similar to the rest-conditions experiment (Figures 7D,E). In Figure 7F, the tracking performance during the rest-to-exercise experiment is illustrated for both controllers. During this experiment, wherein the pump has to provide the major fraction of the CO, the RMSE obtained with the PF-PIDC remained at a level of 1.56 *L min*^−1^, while the maximum error converged to 4.63 *L min*^−1^. The PDD-ILC although showed a reduction in the tracking accuracy, it considerably outperformed the PF-PIDC. More precisely, the RMSE and the maximum error obtained by the PDD-ILC after convergence was 0.68 *L min*^−1^ and 2.46 *L min*^−1^, respectively.

The tracking performance of the controllers under counterpulsation mode is illustrated in Figure 8 for the conducted simulations. During Exp0 (Figure 8A), the PF-PIDC reached an RMSE and maximum error of 0.42 *L min*^−1^ and 1.32 *L min*^−1^, respectively. In this setting, the PDD-ILC required 50 iterations to converge at an RMSE and maximum error of 0.88 *L min*^−1^ and 2.64 *L min*^−1^, although it obtained similar error values already after the thirtieth iteration. During Exp1 (Figure 8B), Exp3 (Figure 8D), and Exp4 (Figure 8E) both controllers obtained tracking errors similar to those in Exp0 after convergence, however, the tracking error was increased during the transition phases of the experiments. During Exp2 both controllers converged to error values lower than Exp0 (Figure 8C). More precisely, the PDD-ILC converged to an RMSE and maximum error of 0.50 *L min*^−1^ and 1.67 *L min*^−1^ and the PF-PIDC to 0.19 *L min*^−1^ and 0.50 *L min*^−1^, respectively. Similar to the copulsation mode, during the rest-to-exercise experiment (Exp5), the tracking error was increased for both controllers. As it can be seen in Figure 8F, the PDD-ILC obtained an RMSE and maximum error of 1.61 *L min*^−1^ and 3.06 *L min*^−1^, outperforming the PF-PIDC that converged to 1.88 *L min*^−1^ and 4.18 *L min*^−1^, respectively.

The addition of noise in the simulated LVP and PF signals had infinitesimal effect on the reference trajectory tracking. In the Supplementary Material, the detailed results for white noise of 0.86 variance can be found in Supplementary Figure S1 for all experiments under copulsation and Supplementary Figure S2 under counterpulsation, while for white noise of 1.72 variance can be found in Supplementary Figure S3 for all experiments under copulsation and Supplementary Figure S4 under counterpulsation.

### Ventricular Unloading, Pulsatility and Hemodynamic Response

**Figure 9:**
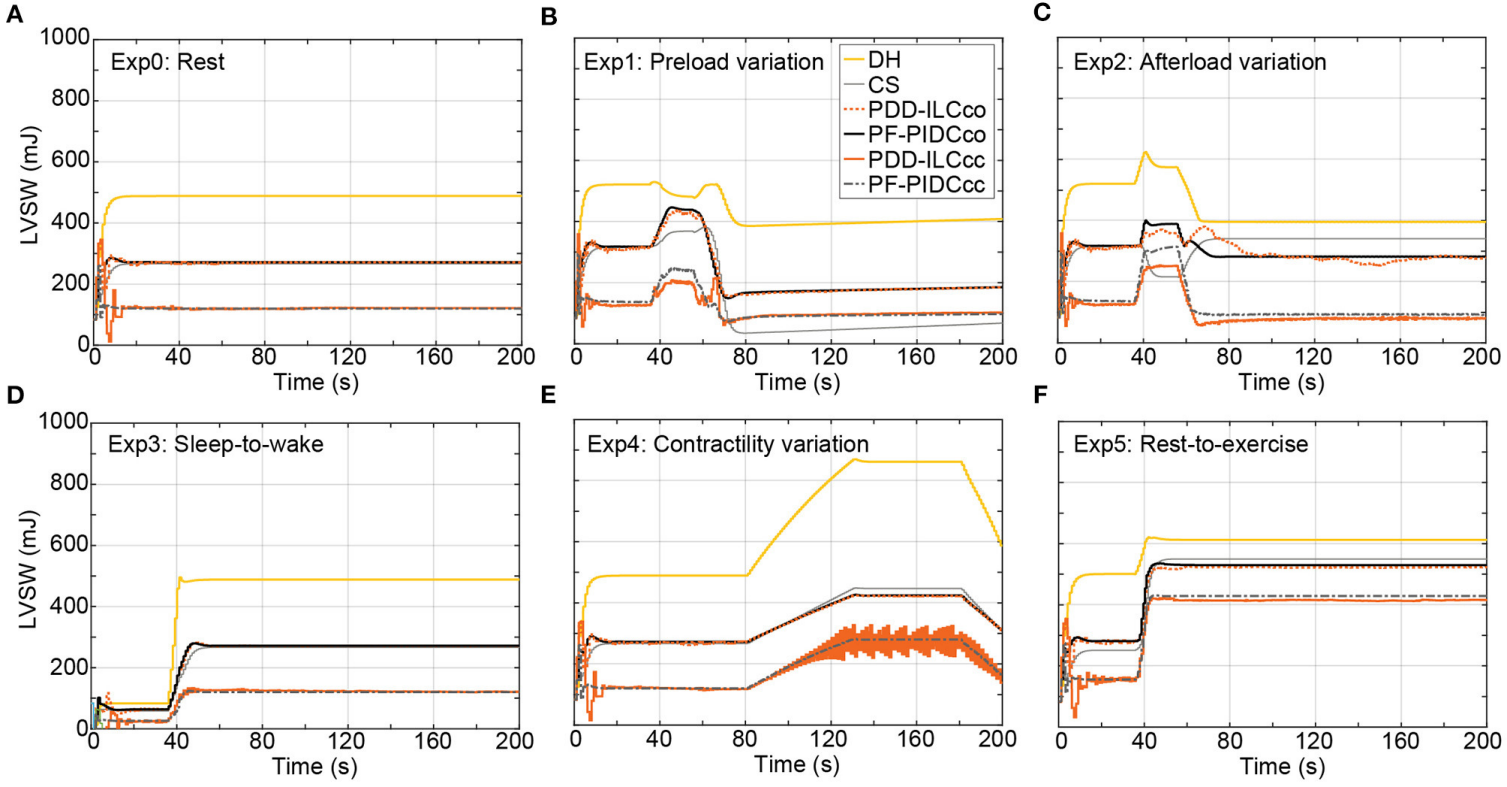
Influence of the copulsation and counterpulsation modes on the left ventricular stroke work (LVSW) of the diseased heart (DH) during: **(A)** Rest-conditions (Exp0), **(B)** Preload variation (Exp1 **(C)** Afterload variation (Exp2) **(D)** Sleep-to-wake (Exp3) **(E)** Contractility variation (Exp4) **(F)** Rest-to-exercise (Exp5). DH, diseased heart; CS, constant speed control; PDD-ILCco, physiologic data-driven iterative learning controller copulsation; PF-PIDCco, pump flow proportional-integral-derivative controller copulsation; PDD-ILCcc, physiologic data-driven iterative learning controller counterpulsation; PF-PIDCcc, pump flow proportional-integral-derivative controller counterpulsation.

In this study, the reference PF trajectories were obtained by using copulsation and counterpulsation as support modes, aiming to increase the pulsatility or reduce the LVSW, respectively. The influence of both modes on the LVSW is illustrated in Figure 9 for the executed experiments and is compared with the LVSW produced by the simulated diseased heart (DH) and the DH supported with a cfVAD with a constant speed controller (CS). During the experiments Exp0 (Figure 9A), Exp3 (Figure 9D), Exp4 (Figure 9E), and Exp5 (Figure 9F), using copulsation mode, both the PDD-ILC and the PF-PIDC controller followed the LVSW values obtained with the CS controller. In the same experiments, under counterpulsation, both the PDD-ILC and the PF-PIDC controller reduced the LVSW by 54.3, 55.9, 69.8, and 24% compared to the CS support. During the preload variation (Figure 9B), the PDD-ILC and the PF-PIDC controller showed similar responses, reducing the LVSW by 56% compared to the CS and by 57.2% compared to the copulsation modes. However, during the low preload conditions, applied after the last transition point of Exp1, the CS reduced the LVSW by 26.3% compared to the PDD-ILC and PF-PIDC controller under counterpulsation. As it can be seen in Figure 9C, during low afterload conditions (between 40 and 80 seconds), the PDD-ILC and the PF-PIDC controller under counterpulsation, as well as the CS controller, obtained similar LVSW values, while the copulsation modes resulted in 31.2% higher LVSW values. However, during the high afterload conditions in Exp 2 (after 80s in Figure 9C), the PDD-ILC and the PF-PIDC controller under counterpulsation resulted in 75.7 and 72.7%, respectively, compared to CS support. Overall, both the PDD-ILC and the PF-PIDC achieved the intended LVSW reduction during counterpulsation modes.

**Figure 10:**
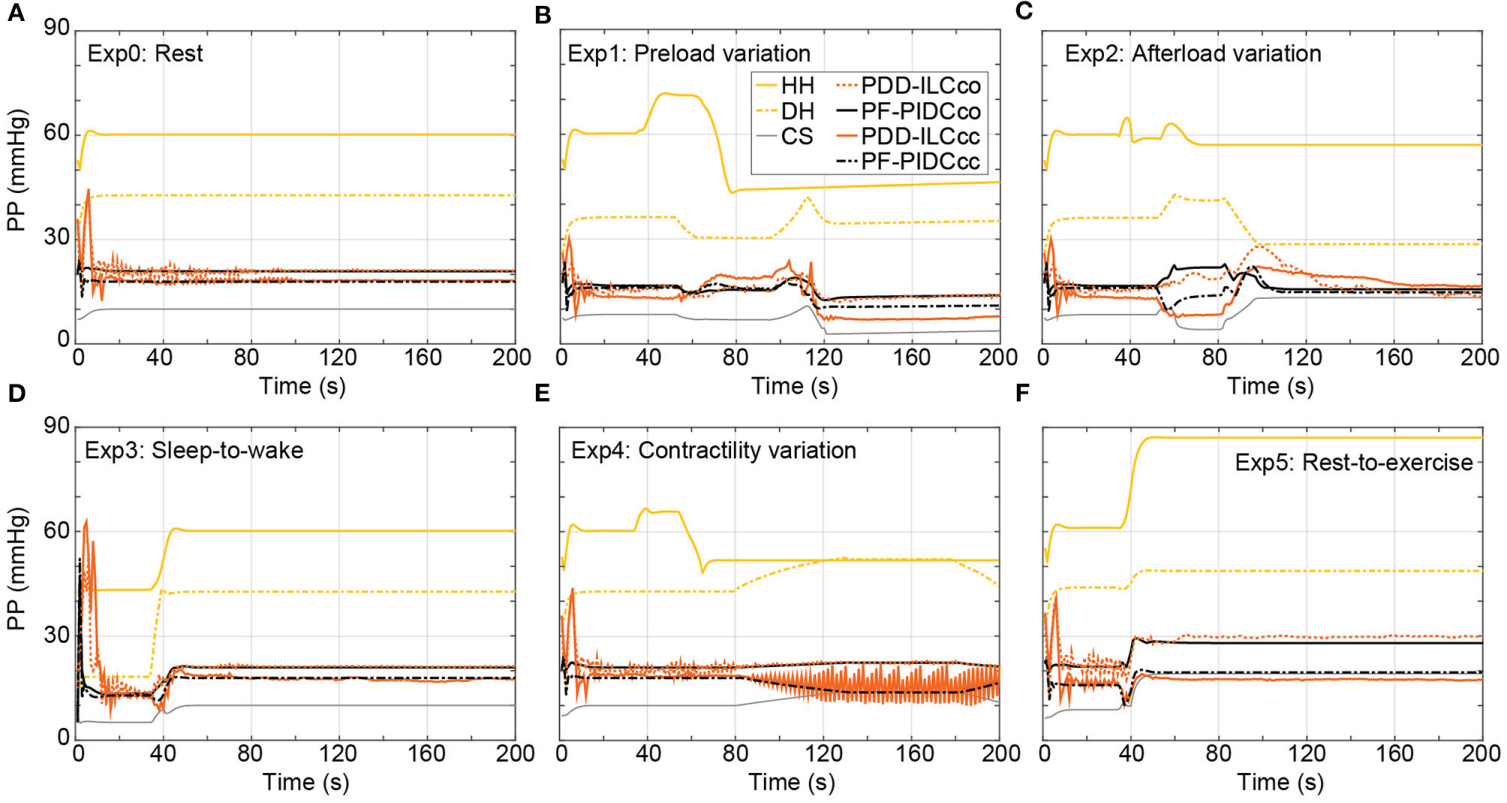
Influence of the copulsation and counterpulsation modes on the aortic pulse pressure (PP) of the diseased heart (DH) during: **(A)** Rest-conditions (Exp0), **(B)** Preload variation (Exp1) **(C)** Afterload variation (Exp2) **(D)** Sleep-to-wake (Exp3) **(E)** Contractility variation (Exp4) **(F)** Rest-to-exercise (Exp5). HH, healthy heart; DH, diseased heart; CS, constant speed control; PF-PIDCco, pump flow proportional-integral-derivative controller copulsation; PDD-ILCcc, physiologic data-driven iterative learning controller counterpulsation; PF-PIDCcc, pump flow proportional-integral-derivative controller counterpulsation.

The influence of the copulsation and the counterpulsation modes on the pulsatility is evaluated based on the aortic pulse pressure (PP = systolic aortic pressure – diastolic aortic pressure) and is illustrated in Figure 10 for all experiments. During Exp0 (Figure 10A) and Exp3 (Figure 10D) the CS diminishes significantly the pulsatility, obtaining a PP of only 10.1 mmHg. Both the PDD-ILC and the PF-PIDC under the counterpulsation mode increased the PP to 16.2 and 17.9 mmHg for Exp0 and Exp3, respectively, while under copulsation, the PDD-ILC and the PF-PIDC further increased the PP to 21.1 and 20.9 mmHg for Exp0 and Exp3, respectively. During the preload experiment (Figure 10B) and after the convergence of all controllers, the CS resulted in the lowest PP of 4.0 mmHg, the PDD-ILC resulted in 14.6 and 15.2 mmHg for counterpulsation and copulsation, respectively, while the PF-PIDC resulted in 9.4 and 11.6 mmHg for counterpulsation and copulsation. During the transition phases of the afterload experiment (Figure 10C), the CS reduced the PP to only 4.1 mmHg, however, the PDD-ILC resulted in significantly increased PP values of 8.4 and 18.7 mmHg with counterpulsation and copulsation, respectively. During the same settings, the PF-PIDC increased further the PP with respect to CS and PDD-ILC, achieving a PP of 13.8 and 29.9 mmHg under counterpulsation and copulsation, respectively. At high afterload conditions in Exp2 (after 110 s in Figure 10C), all controllers resulted in similar PP value of approximately 14.9 mmHg. During the Exp4 (Figure 10E) both the PDD-ILC and the PF-PIDC with copulsating mode resulted in PP of 19.8 mmHg, while the CS, as well as the PDD-ILC and the PF-PIDC under counterpulsation showed reduced pulsatility, obtaining a PP of 10.5, 20.0 and 18.9 mmHg, respectively. The PDD-ILC presented an oscillating PP when the contractility reached 17% of that of the HH. During the rest-to-exercise experiment (Figure 10F), the PDD-ILC with counterpulsation resulted in the lowest PP of 17.5 mmHg, while the CS and the PF-PIDC resulted in 19.5 and 19.1 mmHg. Under the same settings, the PID-controller and the PDD-ILC under copulsation increased significantly the PP achieving 28.0 and 30.0 mmHg, respectively.

### Preload and Afterload Sensitivity

The sensitivity of the developed controllers in preload and afterload changes was evaluated in Exp1 and Exp2, respectively, and it was compared with the sensitivities of the CS controller and the HH. Based on the equations given in Supplementary Text T1 in the Supplementary Material, the end-diastolic LV pressure and the mean aortic pressure (MAP) were used as surrogates of the preload and the afterload, respectively, and a summary of all sensitivities is provided in Table 2. As it can be seen in Table 2, both the PDD-ILC and the PF-PIDC showed physiological sensitivities compared to the HH, while the CS support resulted in highly non-physiological sensitivities in all cases. More precisely, during preload increase the HH showed a sensitivity of 0.502 *L min*^−1^/*mmHg*. The PDD-ILC in copulsation mode followed closely this value, resulting in 0.496 *L min*^−1^/*mmHg*, while the PF-PIDC showed 0.488 *L min*^−1^/*mmHg* and 0.470 *L min*^−1^/*mmHg* under copulsation and counterpulsation, respectively. The PDD-ILC in counterpulsation mode had a lower preload sensitivity of 0.177 *L min*^−1^/*mmHg* and the CS showed a highly non-physiologic sensitivity of 0.039 *L min*^−1^/*mmHg*. Similar to preload increase, during preload decrease the developed controllers followed closely the sensitivity of the HH, while the CS showed again a sensitivity of 0.040 *L min*^−1^/*mmHg*. The sensitivity of the HH was −0.015 *L min*^−1^/*mmHg* and −0.016 *L min*^−1^/*mmHg* during afterload increase and decrease, respectively. The PDD-ILC in copulsation mode showed a sensitivity of −0.019 *L min*^−1^/*mmHg* and −0.024 *L min*^−1^/*mmHg* during afterload increase and decrease, while in counterpulsation showed high sensitivity values of *L min*^−1^/*mmHg* in all afterload changes. The PF-PIDC in copulsation and counterpulsation modes responded with a sensitivity of −0.018 *L min*^−1^/*mmHg* and −0.011 *L min*^−1^/*mmHg* to afterload increase, and with a sensitivity of −0.026 *Lmin*^−1^/*mmHg* and −0.019 *L min*^−1^/*mmHg* to afterload decrease. The CS controller showed more than three times higher afterload response that the HH, resulting in sensitivities of −0.046 *L min*^−1^/*mmHg* and −0.057 *L min*^−1^/*mmHg* to afterload increase and decrease, respectively.

**Table 2:** Preload and afterload sensitivity of the healthy heart (HH), the constant speed (CS) controller, the PDD-ILC, and the PF-PIDC calculated in experiments Exp1 and Exp2.

**System**	**Preload (increase) (*L min*^−1^/*mmHg*)**	**Preload (decrease) (*L min*^−1^/*mmHg*)**	**Afterload (increase) (*L min*^−1^/*mmHg*)**	**Afterload (decrease) (*L min*^−1^/*mmHg*)**
Healthy heart	0.502	0.481	−0.015	−0.016
CS controller	0.039	0.040	−0.046	−0.057
PDD-ILCco	0.496	0.466	−0.019	−0.024
PDD-ILCcc	0.177	0.386	−0.030	−0.030
PF-PIDCco	0.488	0.377	−0.018	−0.011
PF-PIDCcc	0.470	0.411	−0.026	−0.019

## Discussion

In the current work, we presented a data-driven iterative learning physiological controller and a pump flow PID-controller that accurately track predefined pump flow trajectories, aiming to achieve physiological, pulsatile and treatment-driven response of cfVADs. A trajectory generator, which can be incorporated as a standalone block in other cfVAD control approaches, was also developed and by exploiting the LV-EDP it provided preload adaptive reference trajectories. In the case of the PDD-ILC, the reference PF trajectories were tracked by a model-free, data-driven ILC that used the time-sequences of LVP and PF to obtain a model. To the best of our knowledge, this is the first application of such a DD-ILC for cfVAD control. Both control approaches have been extensively tested in an *in-silico* environment under various physiological conditions, including rest, pre- and afterload variations, contractility variations, as well as everyday scenarios like sleep-to-wake and rest-to-exercise. Additionally, two treatment objectives were investigated, termed minimization of LVSW (counterpulsation) and maximization of pulsatility (copulsation). Under all experimental conditions, the PDD-ILC and the PF-PIDC demonstrated highly accurate tracking of the reference PF trajectories, outperforming existing model-based iterative ILC approaches, (40) while they also achieved the predefined treatment objectives and resulted in improved hemodynamics and preload sensitivities compared to a CS controller that is the current state-of-the-art in the clinical practice (Table 2) (49).

The reference trajectories constituted a critical component of the DD-IILPC and the PF-PIDC since they were responsible to provide preload adaptivity and incorporate the treatment objectives. To obtain preload adaptivity, the Frank-Starling mechanism was imitated by selecting the preload sensitivity of the controller in Equation (3). To our knowledge, this is the first time that the preload sensitivity can be directly selected based on clinical input, constituting a great improvement compared to CS controllers and speed-based controllers, where fine-tuning of the control gains is necessary to achieve adequate sensitivity (19, 34, 50). Based on the results in section “Preload and Afterload Sensitivity”, the PDD-ILC and the PF-PIDC were able to follow the set value and provide preload sensitivities similar to the healthy heart, while the CS controller showed infinitesimal sensitivity. Hence, based on our approach, a patient-specific preload sensitivity is feasible for both controllers, offering new opportunities in cfVAD treatment management.

The treatment objectives incorporated in the reference trajectories were the minimization of LVSW and the maximization of pulsatility. To minimize the LVSW, a counterpulsating pump modulation with respect to the native heart has been applied as proposed in the literature (35, 37, 44). By accurately tracking the counterpulsation PF trajectories developed in section “Reference Pump Flow Trajectory Generator”, the PDD-ILC and the PF-PIDC were able to substantially reduce the LVSW by more than 50% compared to the CS support in the majority of the investigated physiological conditions. This is important when treatment approaches for LV training are considered. To maximize the pulsatility, a copulsation trajectory was developed and tracked by the PDD-ILC and the PF-PIDC controller. During all physiological conditions studied, both controllers increased drastically the PP compared to the CS support. These results agree with the literature (35, 37, 44) and, consequently, they can be implemented to enhance the pulsatility and investigate its effects and its necessity on cfVAD supported patients. It is important to mention that the effectiveness of our pipeline in reducing the LVSW and increasing the PP is mainly dependent on the developed PF reference trajectories. The difference in LVSW reduction and PP increase between the PDD-ILC and the PFPIDC are a result of their slightly different tracking performance. By exploiting the accurate trajectory tracking achieved from both the PDD-ILC and the PF-PIDC, various phase shifts can be investigated to deduce a better understanding of the interactions between the cfVAD and the native heart to improve the treatment of heart failure patients.

The tracking performance of the DD-ILC and the PF-PIDC was excellent for the copulsation mode, regardless of the physiological conditions applied and the rapid changes in the hemodynamics and the heartbeat. During the counterpulsation, both controllers demonstrated lower tracking accuracy in all experiments compared to the copulsation. However, they significantly outperformed existing ILC approaches (40). The inferior tracking performance of the controllers under counterpulsation can be attributed to two reasons. Firstly, during the counterpulsation, a rapid change of head pressure is applied on the pump from the heart contraction, which cannot be counteracted by the slower dynamic response of the pump. Secondly, the controller parameters were optimized for the copulsation mode, hence better tracking performance during counterpulsation could be achieved with the further optimization of the control parameters.

The control parameters have a strong influence on the stability and the tracking performance of both the PDD-ILC and the PF-PIDC; hence, their selection is of high importance. In this work, we implemented the genetic-algorithm-based optimization framework proposed by Magkoutas et al. (48) to obtain the optimum parameter sets for our control approaches. By using the latter optimization framework, intuitive tuning of the control parameters can be achieved based on the selection of the objective functions to be minimized. Hence, by exploiting the GAOF, the gains of the PDD-ILC and the PF-PIDC controller can be further optimized to facilitate patient-specific treatment goals and, consequently, enhance the prognosis of cfVAD supported patients. However, it has to be mentioned that, depending on the selected objective functions and the experiments in the optimization process, the development phase of the controller can be prolonged since the completion of the optimization might take up to 20 days.

Although the superiority of the PDD-ILC and the PF-PIDC over the CS controller with respect to hemodynamics and pre- and afterload sensitivities has been demonstrated, the proposed control approaches have also limitations. The development of the reference trajectories assumes negligible flow through the aortic valve, however, when a phase shift between the pump pulsation and the native heart has to be incorporated in the reference trajectory (e.g., counterpulsation) the assumption of negligible flow through the aortic valve is violated. This affects the development of a feasible PF trajectory. Additionally, the assumption of negligible aortic valve flow results in reference trajectories that aim to achieve the necessary CO only through the cfVAD operation. Consequently, the flow through the aortic valve is minimized and the risk of aortic valve insufficiency increases. Although such pathological consequences were not within the scope of this study, to ameliorate the risk of aortic valve insufficiency we envisage the addition of a support level parameter to manipulate the percentage of the CO delivered by the pump and the CO expected from the remaining contraction of the native heart.

The excellent tracking performance of both the PDD-ILC and the PF-PIDC necessitates the accurate measurement of LVP and PF signals. We are aware that no reliable, long-term blood pressure and flow sensors are currently available for cfVADs; however, the approach developed by von Petersdorff-Campen et al. (43) is promising and could pave the way toward the realization of LVP and PF measurements. In this study, we have accounted for the inherent noise of real measurement and its effect on the tracking performance by assessing the PDD-ILC and the PF-PIDC tracking ability when white noise was added on the LVP and the PF signals. Additionally, the overall performance of the proposed control approaches has been evaluated only in an *in-silico* environment with a numerical model of a non-implantable mixed-flow turbodynamic blood pump. *In-silico*, studies with the numerical model of the current state of the art blood pump HeartMate 3 have to be also performed. Furthermore, to prove the performance of the controllers in the real-time setting and allow the translation of the controllers into the clinical practice, *in-vitro* and *in-vivo* studies have to be conducted.

Finally, considering the high complexity of the presented control schemes, suction prevention features were not included in the main control structures. Although no suction events were identified in the executed experiments, safety controllers similar to those proposed by Petrou et al. (34) could be incorporated.

## Data Availability Statement

The raw data supporting the conclusions of this article will be made available by the authors, without undue reservation.

## Author Contributions

The study was performed at the Product Development Group Zurich, ETH Zurich, Zurich. The control schemes and the evaluation experiments were conceived and designed by KM and PA. Data collection and analysis was performed by KM and PA. Literature search and study discussions conducted by KM, AP, and MS. The manuscript was prepared by KM and reviewed by PA, MS, and MM. All authors contributed to the article and approved the submitted version.

## Funding

This work was supported by Stavros Niarchos Foundation (SNF). Open access funding provided by ETH Zurich.

## Conflict of Interest

The authors declare that the research was conducted in the absence of any commercial or financial relationships that could be construed as a potential conflict of interest.

## Publisher's Note

All claims expressed in this article are solely those of the authors and do not necessarily represent those of their affiliated organizations, or those of the publisher, the editors and the reviewers. Any product that may be evaluated in this article, or claim that may be made by its manufacturer, is not guaranteed or endorsed by the publisher.
