# Supplementary material for: Physiologic Data-Driven Iterative Learning Control for Left Ventricular Assist Devices
Source: Front Cardiovasc Med. 2022 Jul 13;9:922387. doi: 10.3389/fcvm.2022.922387 (PMC9326058; doi:10.3389/fcvm.2022.922387)
Supplement: Supplementary file 1 [file Data_Sheet_1.docx]

Physiologic Data-Driven Iterative Learning Control for Left Ventricular Assist Devices

Konstantinos Magkoutas^1†^, Philip Arm^1†^, Mirko Meboldt^1^, Marianne Schmid Daners^1*^

^†^These authors have contributed equally to this work

^1^Product Development Group Zurich, Department of Mechanical and Process Engineering, ETH Zurich, Zurich, Switzerland

*** Correspondence:**Marianne Schmid Daners

marischm@ethz.ch

Keywords: LVAD, Heart failure, Data driven control, Iterative learning control, VAD physiological control, Ventricular assist devices, ILC, pulsatile blood pump

# **Abstract**

Continuous flow ventricular assist devices (cfVADs) constitute a viable and increasingly used therapy for end-stage heart failure patients. However, they are still operating at a fixed-speed mode that precludes physiological cfVAD response and it is often related to adverse events of cfVAD therapy. To ameliorate this, various physiological controllers have been proposed, however, the majority of these controllers do not account for the lack of pulsatility in the cfVAD operation, which is supposed to be beneficial for the physiological function of the cardiovascular system. In this study, we present a physiological data-driven iterative learning controller (PDD-ILC) that accurately tracks predefined pump flow trajectories, aiming to achieve physiological, pulsatile, and treatment-driven response of cfVADs. The controller has been extensively tested in an in-silico environment under various physiological conditions, and compared with a physiological pump flow proportional-integral-derivative controller (PF-PIDC) developed in this study as well as the constant speed (CS) control that is the current state of the art in clinical practice. Additionally, two treatment objectives were investigated to achieve pulsatility maximization and left ventricular stroke work (LVSW) minimization by implementing copulsation and counterpulsation pump modes, respectively. Under all experimental conditions, the PDD-ILC as well as the PF-PIDC demonstrated highly accurate tracking of the reference pump flow trajectories, outperforming existing model-based iterative learning control approaches. Additionally, the developed controllers achieved the predefined treatment objectives and resulted in improved hemodynamics and preload sensitivities compared to the CS support.

Supplementary Material

Table of Contents

[**Abstract** 1](#_Toc105880749)

[**Table S1:** 3](#_Toc105880750)

[**Table S2:** 4](#_Toc105880751)

[**Table S3:** 5](#_Toc105880752)

[**Table S4:** 6](#_Toc105880753)

[**Figure S1.** 7](#_Toc105880754)

[**Figure S2.** 8](#_Toc105880755)

[**Figure S3.** 9](#_Toc105880756)

[**Figure S4.** 10](#_Toc105880757)

[**Figure S5.** 11](#_Toc105880758)

[**Figure S6.** 12](#_Toc105880759)

[**Figure S7.** 13](#_Toc105880760)

[**Figure S8.** 14](#_Toc105880761)

[**Figure S9.** 15](#_Toc105880762)

[**Text T1. Preload and afterload sensitivities** 16](#_Toc105880763)

**Table S1:** Parameter sets of the experiments used in the study. SVR, systemic vascular resistance; PVR, pulmonary vascular resistance; UVV, unstressed venous volume; HR, heart rate; LVP, left ventricular pressure; PF, pump flow. The contractility is expressed as a percentage of the healthy heart value.

| Experiment | Scenario | Time [s] | SVR [mmHgs/mL] | PVR [mmHgs/mL] | UVV [ml] | HR [bpm] | Contractility [%] | Noise LVP | Noise PF |
| --- | --- | --- | --- | --- | --- | --- | --- | --- | --- |
| Exp0 | Rest | [0 200] | 1.11 | 0.1 | 2520 | 60 | 34 | 0 | 0 |
| Exp1 | Preload variation | [0, 35, 40, 55, 65, 200] | 1.11 | 0.1 | [2520, 2520, 2270, 2270, 2720, 2720] | 90 | 34 | 0 | 0 |
| Exp2 | Afterload variation | [0, 35, 40, 55, 65, 200] | [1.11, 1.11, 0.51, 0.51, 1.91, 1.19] | 0.1 | 2520 | 90 | 34 | 0 | 0 |
| Exp3 | Sleep to wake | [0, 35, 40, 200] | [1.65, 1.65, 1.11, 1.11] | 0.1 | [2740, 2740, 2520, 2520] | 90 | 34 | 0 | 0 |
| Exp4 | Contractility variation | [0, 50, 100, 150, 200] | 1.11 | 0.1 | 2520 | 90 | [34, 34, 51, 51, 17] | 0 | 0 |
| Exp5 | Rest to exercise | [0, 35, 40, 200] | [0.98, 0.98, 0.5, 0.5] | [0.08, 0.08, 0.03, 0.03] | [2520, 2520, 2020, 2020] | [60 60 80 80] | 34 | 0 | 0 |

**Table S2:** Parameter sets of the experiments used in the study. SVR, systemic vascular resistance; PVR, pulmonary vascular resistance; UVV, unstressed venous volume; HR, heart rate; LVP, left ventricular pressure; PF, pump flow. The contractility is expressed as a percentage of the healthy heart value.

| Experiment | Scenario | Time [s] | SVR [mmHgs/mL] | PVR [mmHgs/mL] | UVV [ml] | HR [bpm] | Contractility [%] | Noise LVP | Noise PF |
| --- | --- | --- | --- | --- | --- | --- | --- | --- | --- |
| Exp0n | Rest | [0 200] | 1.11 | 0.1 | 2520 | 60 | 34 | 0.86 | 0.86 |
| Exp1n | Preload variation | [0, 35, 40, 55, 65, 200] | 1.11 | 0.1 | [2520, 2520, 2270, 2270, 2720, 2720] | 90 | 34 | 0.86 | 0.86 |
| Exp2n | Afterload variation | [0, 35, 40, 55, 65, 200] | [1.11, 1.11, 0.51, 0.51, 1.91, 1.19] | 0.1 | 2520 | 90 | 34 | 0.86 | 0.86 |
| Exp3n | Sleep to wake | [0, 35, 40, 200] | [1.65, 1.65, 1.11, 1.11] | 0.1 | [2740, 2740, 2520, 2520] | 90 | 34 | 0.86 | 0.86 |
| Exp4n | Contractility variation | [0, 50, 100, 150, 200] | 1.11 | 0.1 | 2520 | 90 | [34, 34, 51, 51, 17] | 0.86 | 0.86 |
| Exp5n | Rest to exercise | [0, 35, 40, 200] | [0.98, 0.98, 0.5, 0.5] | [0.08, 0.08, 0.03, 0.03] | [2520, 2520, 2020, 2020] | [60 60 80 80] | 34 | 0.86 | 0.86 |

**Table S3:** Parameter sets of the experiments used in the study. SVR, systemic vascular resistance; PVR, pulmonary vascular resistance; UVV, unstressed venous volume; HR, heart rate; LVP, left ventricular pressure; PF, pump flow. The contractility is expressed as a percentage of the healthy heart value.

| Experiment | Scenario | Time [s] | SVR [mmHgs/mL] | PVR [mmHgs/mL] | UVV [ml] | HR [bpm] | Contractility [%] | Noise LVP | Noise PF |
| --- | --- | --- | --- | --- | --- | --- | --- | --- | --- |
| Exp0nn | Rest | [0 200] | 1.11 | 0.1 | 2520 | 60 | 34 | 1.72 | 1.72 |
| Exp1nn | Preload variation | [0, 35, 40, 55, 65, 200] | 1.11 | 0.1 | [2520, 2520, 2270, 2270, 2720, 2720] | 90 | 34 | 1.72 | 1.72 |
| Exp2nn | Afterload variation | [0, 35, 40, 55, 65, 200] | [1.11, 1.11, 0.51, 0.51, 1.91, 1.19] | 0.1 | 2520 | 90 | 34 | 1.72 | 1.72 |
| Exp3nn | Sleep to wake | [0, 35, 40, 200] | [1.65, 1.65, 1.11, 1.11] | 0.1 | [2740, 2740, 2520, 2520] | 90 | 34 | 1.72 | 1.72 |
| Exp4nn | Contractility variation | [0, 50, 100, 150, 200] | 1.11 | 0.1 | 2520 | 90 | [34, 34, 51, 51, 17] | 1.72 | 1.72 |
| Exp5nn | Rest to exercise | [0, 35, 40, 200] | [0.98, 0.98, 0.5, 0.5] | [0.08, 0.08, 0.03, 0.03] | [2520, 2520, 2020, 2020] | [60 60 80 80] | 34 | 1.72 | 1.72 |

**Table S4:** Upper and lower limits of the control parameters of the PDD-ILC and the PF-PIDC used in the minimization problem of the genetic algorithm-based optimization framework.

| PDD-ILC | | | PF-PIDC | | |
| --- | --- | --- | --- | --- | --- |
| Parameter | Minimum | Maximum | Parameter | Minimum | Maximum |
| $\mu$ | 0 | 2 | $K_{P}$ | 0 | 1000 |
| $\eta$ | 0 | 1 | $K_{I}$ | 0 | 100 |
| $Q$ | 0 | 600 | $K_{D}$ | 0 | 100 |
| $R$ | 0 | 1 |  |  |  |
| $k_{p}$ | 0 | 7 |  |  |  |
| $k_{d}$ | 0 | 7 |  |  |  |


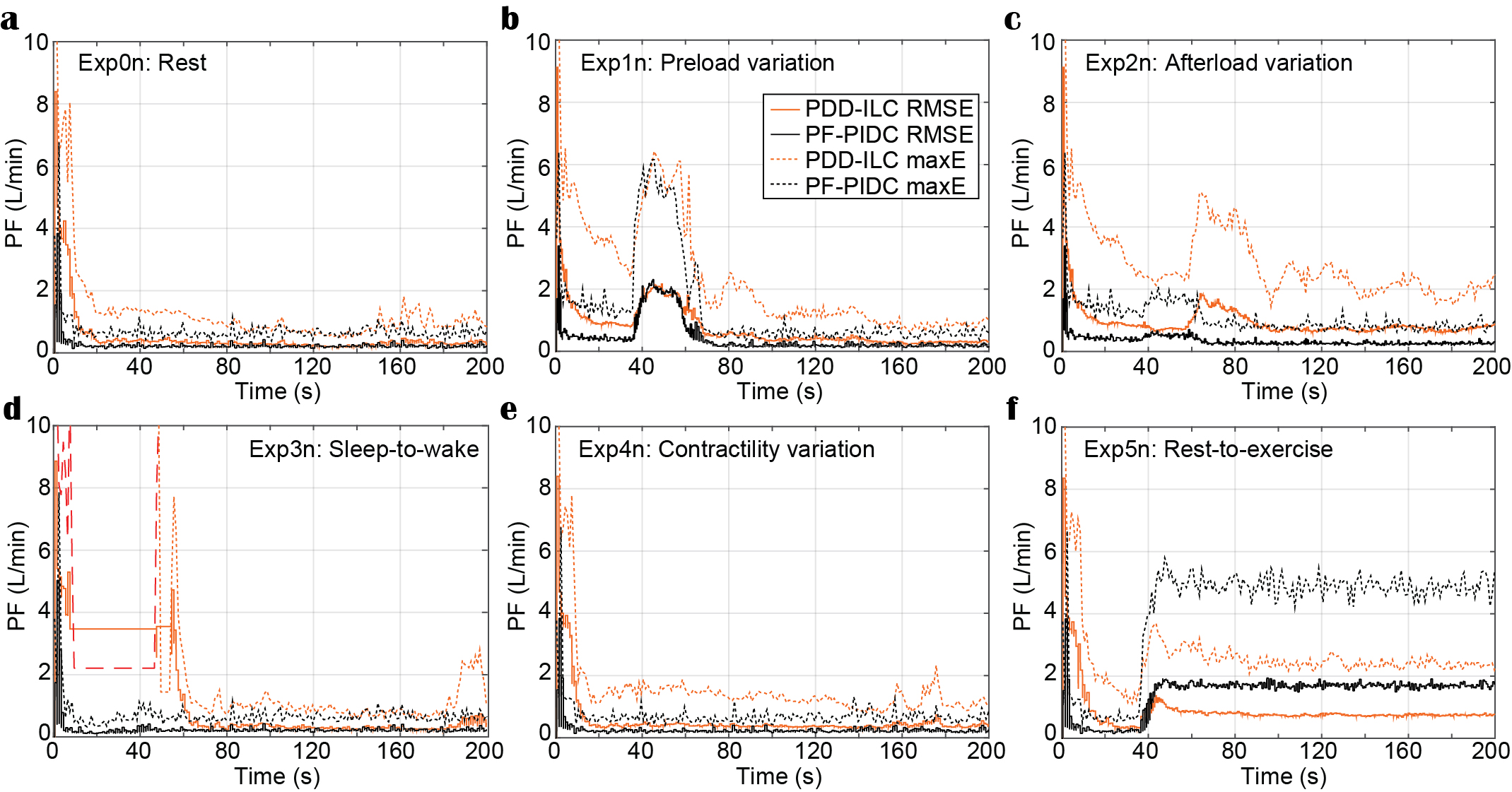


**Figure S1.** Transient performance of the PDD-ILC and the PFPIDC in terms of RMSE and maximum instantaneous error in tracking the reference trajectory under all physiological conditions and scenarios executed with the copulsation mode and white noise of 0.86 variance added on the left ventricular pressure and pump flow signals. **a**) Rest-conditions (Exp0n) **b**) Preload variation (Exp1n) **c**) Afterload variation (Exp2n) **d**) Sleep-to-wake (Exp3n) **e**) Contractility variation (Exp4n) **f**) Rest-to-exercise (Exp5n). *RMSE, root mean square error; maxE, maximum error; PDD-ILC, physiologic data-driven iterative learning controller; PF-PIDC, pump flow proportional-integral-derivative controller.*


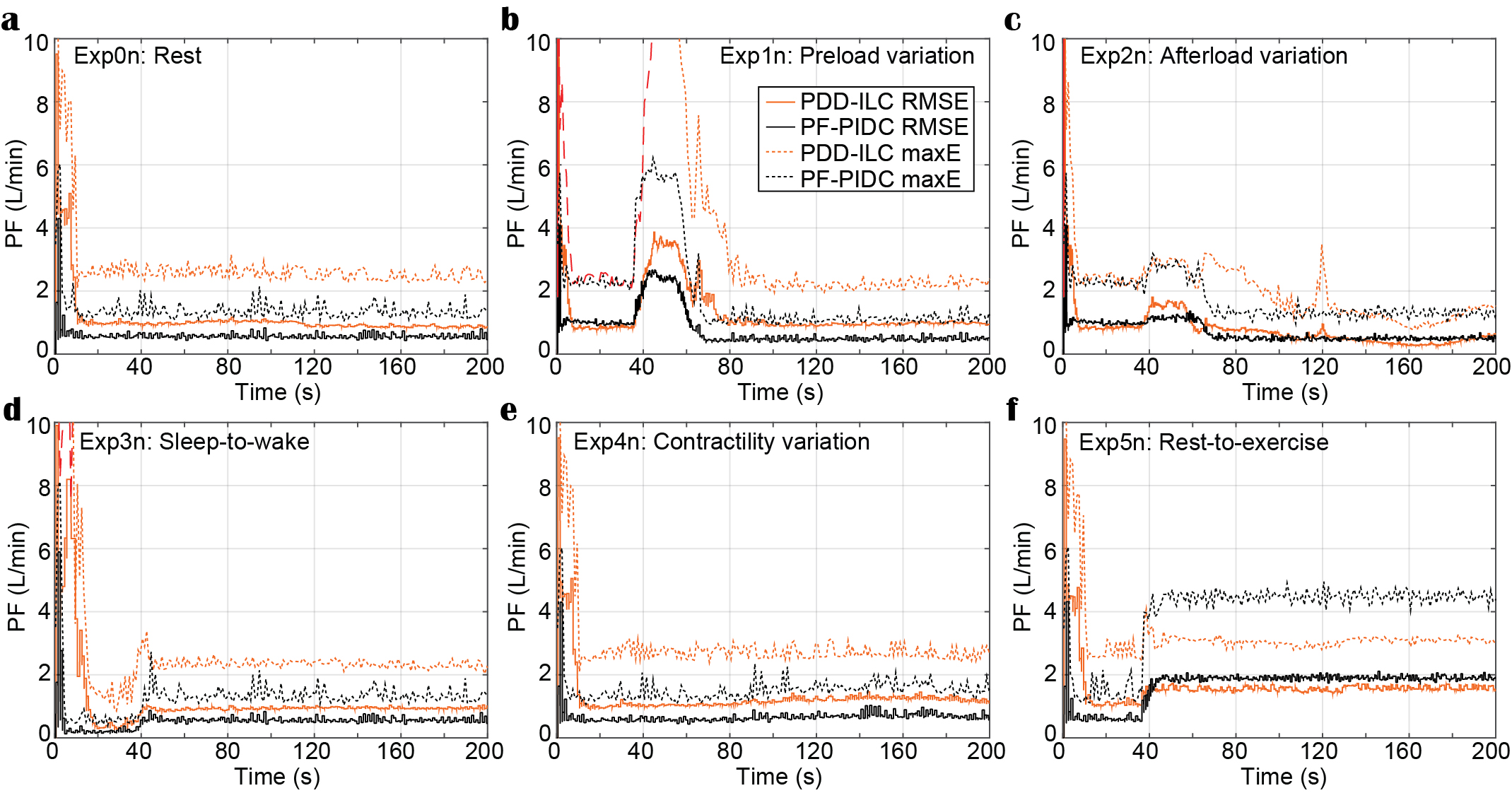


**Figure S2.** Transient performance of the PDD-ILC and the PFPIDC in terms of RMSE and maximum instantaneous error in tracking the reference trajectory under all physiological conditions and scenarios executed with the counterpulsation mode and white noise of 0.86 variance added on the left ventricular pressure and pump flow signals. **a**) Rest-conditions (Exp0n) **b**) Preload variation (Exp1n) **c**) Afterload variation (Exp2n) **d**) Sleep-to-wake (Exp3n) **e**) Contractility variation (Exp4n) **f**) Rest-to-exercise (Exp5n). *RMSE, root mean square error; maxE, maximum error; PDD-ILC, physiologic data-driven iterative learning controller; PF-PIDC, pump flow proportional-integral-derivative controller.*


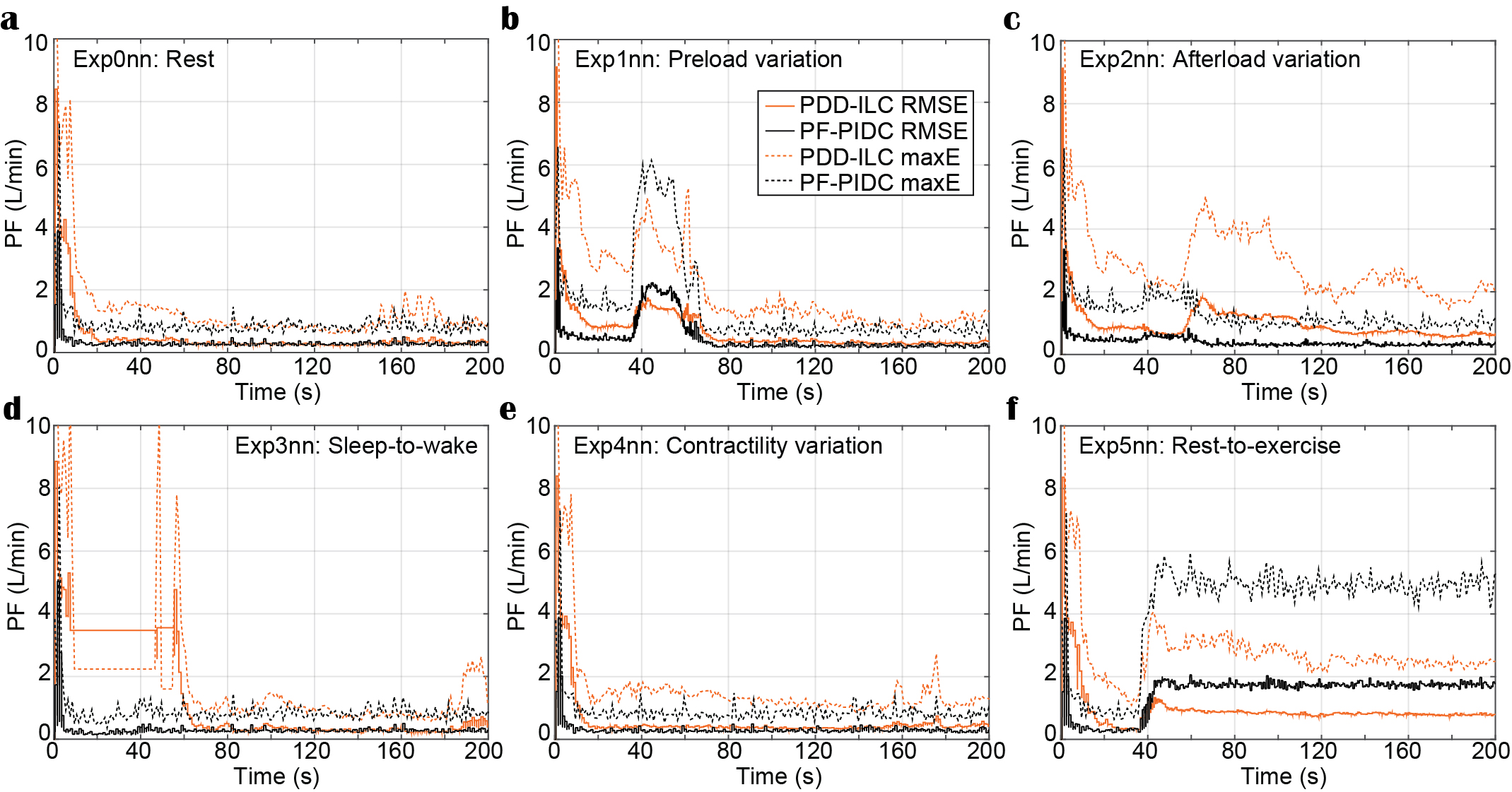


**Figure S3.** Transient performance of the PDD-ILC and the PFPIDC in terms of RMSE and maximum instantaneous error in tracking the reference trajectory under all physiological conditions and scenarios executed with the copulsation mode and white noise of 1.72 variance added on the left ventricular pressure and pump flow signals. **a**) Rest-conditions (Exp0n) **b**) Preload variation (Exp1n) **c**) Afterload variation (Exp2n) **d**) Sleep-to-wake (Exp3n) **e**) Contractility variation (Exp4n) **f**) Rest-to-exercise (Exp5n). *RMSE, root mean square error; maxE, maximum error; PDD-ILC, physiologic data-driven iterative learning controller; PF-PIDC, pump flow proportional-integral-derivative controller.*


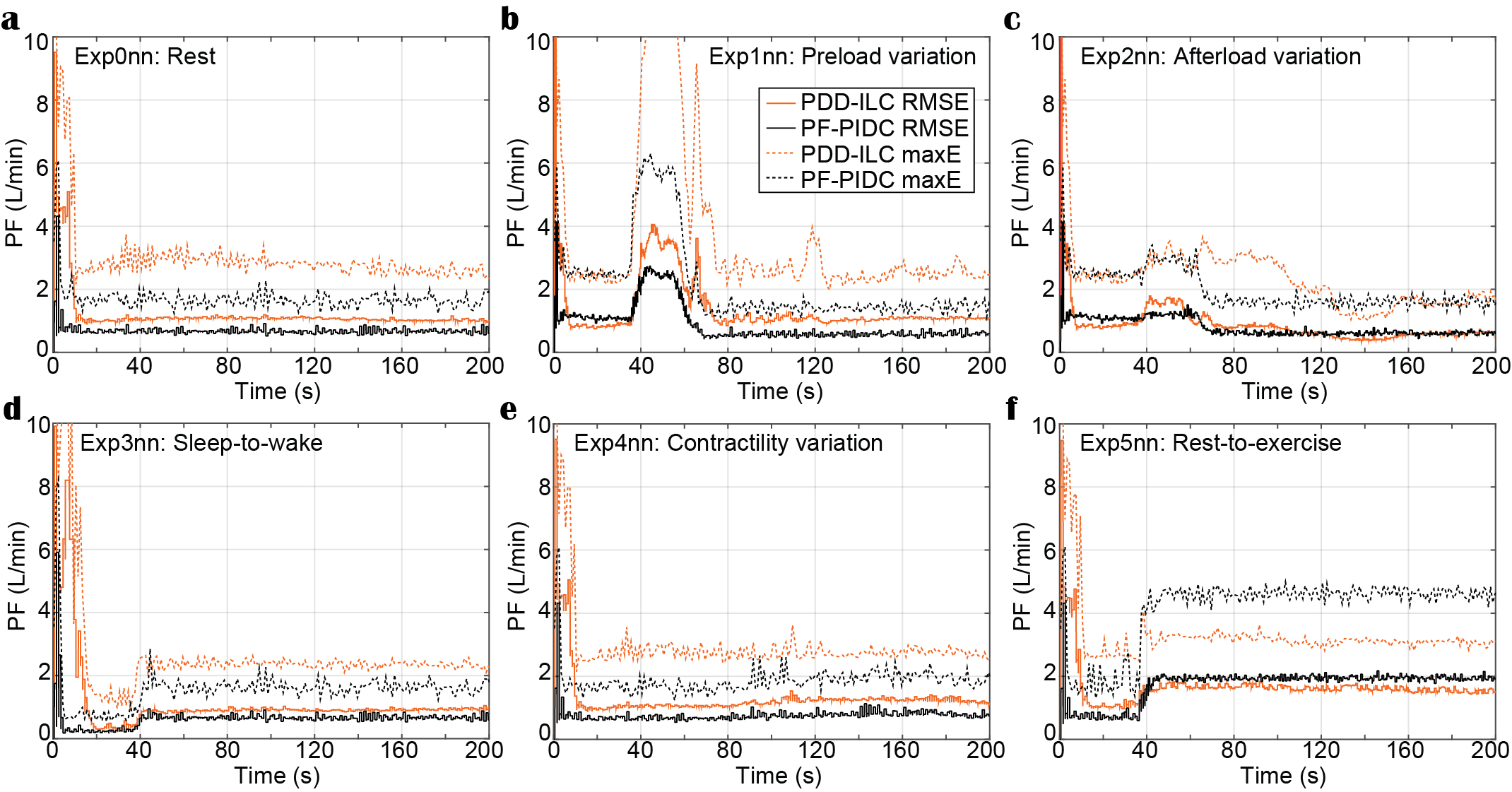


**Figure S4.** Transient performance of the PDD-ILC and the PFPIDC in terms of RMSE and maximum instantaneous error in tracking the reference trajectory under all physiological conditions and scenarios executed with the counterpulsation mode and white noise of 1.72 variance added on the left ventricular pressure and pump flow signals. **a**) Rest-conditions (Exp0n) **b**) Preload variation (Exp1n) **c**) Afterload variation (Exp2n) **d**) Sleep-to-wake (Exp3n) **e**) Contractility variation (Exp4n) **f**) Rest-to-exercise (Exp5n). *RMSE, root mean square error; maxE, maximum error; PDD-ILC, physiologic data-driven iterative learning controller; PF-PIDC, pump flow proportional-integral-derivative controller.*

**
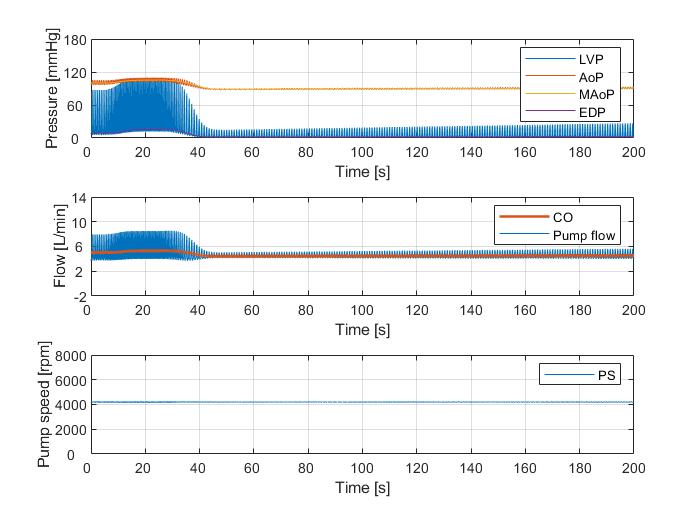
**

**Figure S5.** Hemodynamic and pump parameters during the Preload variation (Exp1) with the diseased heart being supported with a ventricular assist device operating with the constant speed controller. *LVP, left ventricular pressure; AoP, arterial pressure; MAoP, mean arterial pressure; EDP, end diastolic pressure; CO, cardiac output; PS, pump speed.*

**
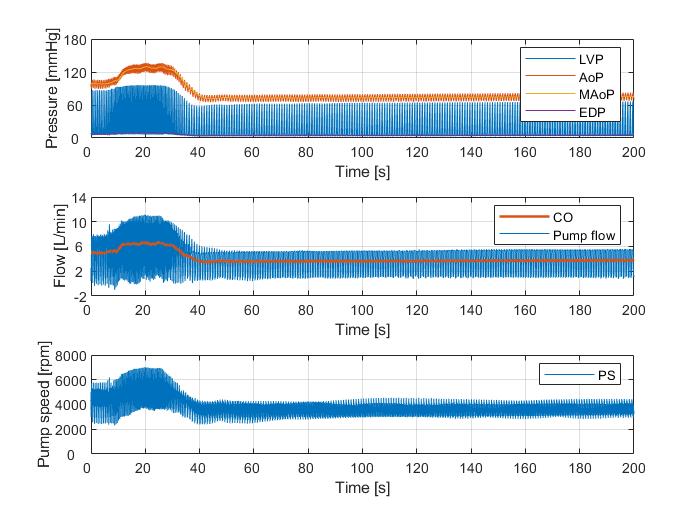
**

**Figure S6.** Hemodynamic and pump parameters during the Preload variation (Exp1) with the diseased heart being supported with a ventricular assist device operating with the PDD-ILC under copulsation mode. *LVP, left ventricular pressure; AoP, arterial pressure; MAoP, mean arterial pressure; EDP, end diastolic pressure; CO, cardiac output; PS, pump speed.*

**
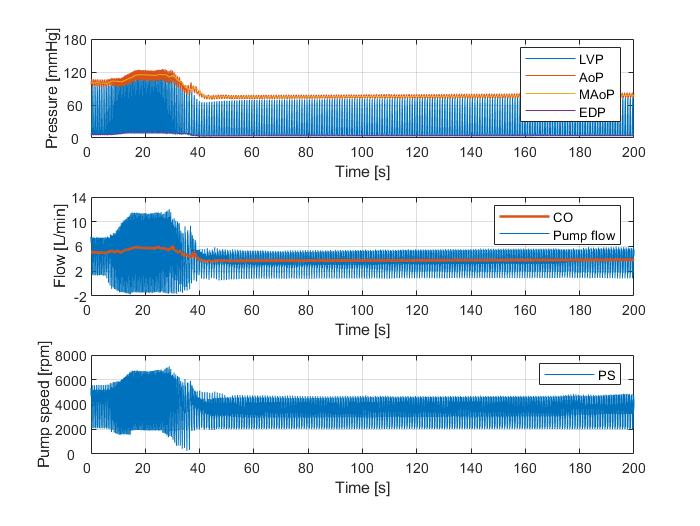
**

**Figure S7.** Hemodynamic and pump parameters during the Preload variation (Exp1) with the diseased heart being supported with a ventricular assist device operating with the PDD-ILC under counterpulsation mode. *LVP, left ventricular pressure; AoP, arterial pressure; MAoP, mean arterial pressure; EDP, end diastolic pressure; CO, cardiac output; PS, pump speed.*

**
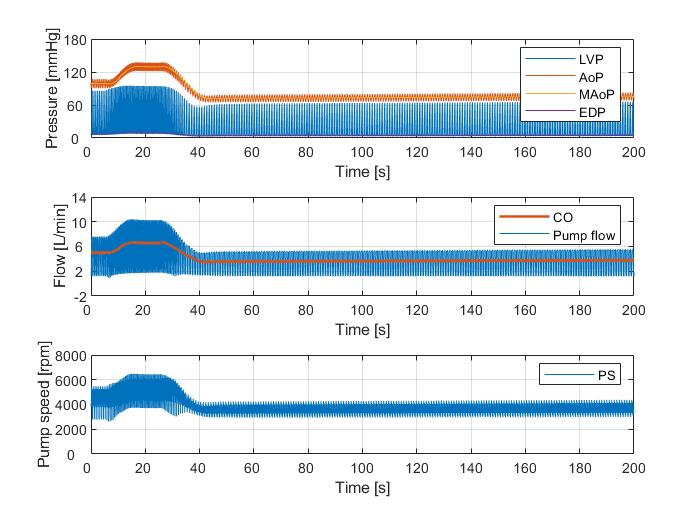
**

**Figure S8.** Hemodynamic and pump parameters during the Preload variation (Exp1) with the diseased heart being supported with a ventricular assist device operating with the PFPIDC under copulsation mode. *LVP, left ventricular pressure; AoP, arterial pressure; MAoP, mean arterial pressure; EDP, end diastolic pressure; CO, cardiac output; PS, pump speed.*

**
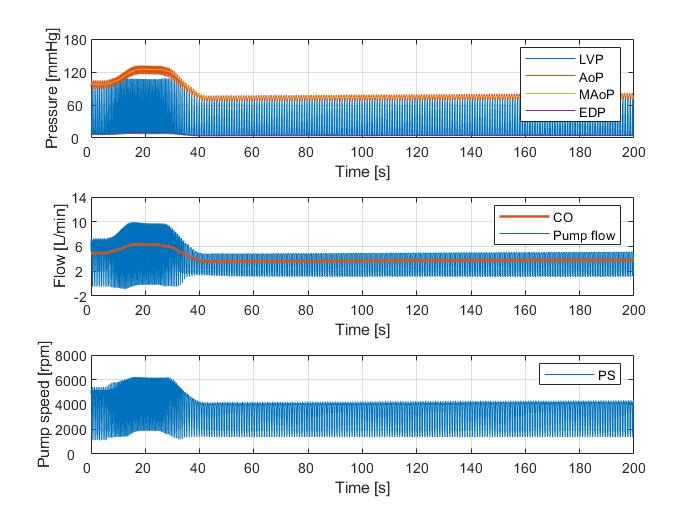
**

**Figure S9.** Hemodynamic and pump parameters during the Preload variation (Exp1) with the diseased heart being supported with a ventricular assist device operating with the PFPIDC under counterpulsation mode. *LVP, left ventricular pressure; AoP, arterial pressure; MAoP, mean arterial pressure; EDP, end diastolic pressure; CO, cardiac output; PS, pump speed.*

**Text T1. Preload and afterload sensitivities**

The preload (afterload) sensitivity of the native heart is defined as the change in the cardiac output (CO) for a given change in the preload (afterload). The definition of the preload and afterload sensitivity of a diseased heart supported by a ventricular assist device (VAD) is the same, however, in this case the CO is calculated as the summation of the aortic valve flow and the flow at the pump outlet.

In this work, the sensitivity of the healthy heart, as well as, the diseased heart supported by a VAD controlled with the developed controllers or under constant speed operation, in preload and afterload changes was evaluated in Exp1 and Exp2, respectively. Specifically, the end-diastolic left ventricular pressure (EDLVP) and the mean aortic pressure (MAP) were used as surrogates of the preload and the afterload. Hence, the preload and afterload sensitivities were calculated as:

$$S_{preload}=\frac{{CO}_{ac}-{CO}_{bc}}{\mathrm{EDLVP}_{ac}-\mathrm{EDLVP}_{bc}} , S_{afterload}=\frac{{CO}_{ac}-{CO}_{bc}}{\mathrm{MAP}_{ac}-\mathrm{MAP}_{bc}}$$

Where $S$ is the sensitivity. The subscript $ac$ defines the value after the applied preload/afterload change while the subscript$bc$ defines the value before the applied preload/afterload change. As mentioned above, the CO equals the aortic valve flow in the case of the healthy heart, while in the case of the diseased heart supported by a VAD, the CO equals the summation of the aortic valve flow and the pump output flow.
